# Early Prediction of Septic Shock in Emergency Department Using Serum Metabolites

**DOI:** 10.1021/jasms.5c00009

**Published:** 2025-05-09

**Authors:** Yu Hong, Li-Hua Li, Ting-Hao Kuo, Yi-Tzu Lee, Cheng-Chih Hsu

**Affiliations:** † Department of Chemistry, 33561 National Taiwan University, 10617, Taipei, Taiwan; ‡ Department of Pathology and Laboratory Medicine, 46615 Taipei Veterans General Hospital, 11217, Taipei, Taiwan; § Ph.D. Program of Medical Biotechnology, Taipei Medical University, 110301, Taipei, Taiwan; ∥ 9471 European Molecular Biology Laboratory, 69117, Heidelberg, Baden-Württemberg, Germany; ⊥ Department of Emergency Medicine, 46615 Taipei Veterans General Hospital, 11217, Taipei, Taiwan; # Faculty of Medicine, School of Medicine, National Yang Ming Chiao Tung University, 112304, Taipei, Taiwan; ∇ Leeuwenhoek Laboratories Co. Ltd, 106070, Taipei, Taiwan

**Keywords:** septic shock, emergency department, prediction, metabolomics, biomarkers, machine learning

## Abstract

Early recognition of septic shock is crucial for improving clinical management and patient outcomes, especially in the emergency department (ED). This study conducted serum metabolomic profiling on ED patients diagnosed with septic shock (n = 32) and those without septic shock (n = 92) using a high-resolution mass spectrometer. By implementing a supervised machine learning algorithm, a prediction model based on a panel of metabolites achieved an accuracy of 87.8%. Notably, when employed on a low-resolution instrument, the model maintained its predictive performance with an accuracy of 84.2%. These results demonstrate the potential of metabolite-based algorithms to identify patients at high risk of septic shock. Our proposed workflow aims to optimize risk assessment and streamline clinical management processes in the ED, holding promise as an efficient routine test to promote timely intensive interventions and reduce septic shock mortality.

## Graphical abstract

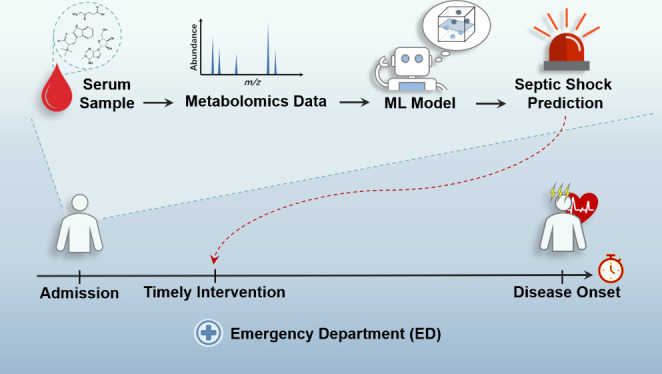

## Introduction

Septic shock, the most severe form of sepsis, arises from a dysregulated host response to infection and leads to fatal multiple organ failures.^1^ Sepsis, including septic shock, is the leading cause of in-hospital deaths and imposes an annual economic burden exceeding US$24 billion in the USA.^2,3^ Globally, sepsis affected 48.9 million people and caused 11 million deaths, accounting for 19.7% of all global deaths in 2017.^4^ Among all septic patients, septic shock has the highest mortality, ranging from 34.3% to 56.7%.^5–8^ Delays in treatment correlate with increased mortality risks.^9^ Consequently, several time-based bundled therapies have become standard practice for treating septic patients,^9–12^ focusing on shortening the time frame for initiating supportive treatment and antibiotic therapy. Early intervention indeed benefits septic shock patients; however, treating all suspected cases leads to overtreatment, such as unnecessary antibiotic prescriptions, aggravating the risk of drug resistance.^13,14^ Within the existing treatment framework, the main challenge is to identify septic shock patients quickly, who are at high risk but could benefit from early intervention.

Early identification of septic shock for immediate intervention is crucial for providing high-quality sepsis care, especially in the emergency department (ED). EDs serve as primary entry points for hospital admission, where patients undergo initial assessment and treatment before being allocated to appropriate levels of care..^15–17^ About one-third of sepsis patients are admitted through the ED,^18^ which means treatment must be initiated here to optimize outcomes. Patients with septic shock, a critical condition necessitating mechanical ventilation, need prompt transfer to specialized units with adequate monitoring capabilities, such as intensive care units. Considering the limited ICU capacity, a tool for early stratification of septic shock patients in the ED is urgently needed to facilitate timely medical interventions and optimize resource allocation.

However, current diagnostics face limitations in achieving rapid and reliable screening for septic shock in the ED. According to the Third International Consensus Definition for Sepsis and Septic Shock (Sepsis-3),^1^ septic shock is defined by an acute increase of the Sequential Organ Failure Assessment (SOFA) score ≥ 2 from baseline, along with the requirement for vasopressors in the setting of elevated lactate. While the SOFA score aims to evaluate several organ systems through a limited number of objective indicators, its utilization is hindered by complex evaluation processes and delays in diagnosis. Moreover, some tests in the score may not be available in basic clinical settings such as EDs.^19^ In contrast, the quick SOFA (qSOFA) score designed for sepsis screening outside the intensive care unit (ICU) provides practicality for ED because it does not require laboratory tests,^1^ but it lacks specificity for septic shock.^19–21^ Despite efforts to utilize common infection-associated biomarkers like lactate, C-reactive protein (CRP), and procalcitonin (PCT) for early detection of septic shock,^22–25^ their individual performance remains limited, with AUC ranging from 0.556 to 0.780.^26,27^ Given the heterogeneous nature of septic shock, it is difficult for a single biomarker to fully reflect the status of potential septic patients. Thus, combining multiple biomarkers with physiological indicators has shown improved performance, with AUC ranging from 0.770 to 0.838.^26,28^ Nevertheless, the medical-economic effect of combining multiple laboratory tests remains controversial, warranting further investigation..^25^

Metabolomics offers a promising avenue for developing clinically practical diagnostic tools. Metabolites serve as crucial indicators of sepsis, revealing downstream products of gene and protein expression.^29–31^ Significantly, metabolic changes often precede the onset of disease symptoms, highlighting their potential as early biomarkers. While metabolomics has been utilized in diagnostic tool development for sepsis-related conditions like infection, organ failure, and mortality,^32–34^ its application specifically for predicting septic shock in the ED setting has yet to be fully discovered. Technologically, metabolomics analysis enables the measurement of a wide range of analytes, thereby facilitating comprehensive investigation of disease pathogenesis pathways.^29,35^ With advances in high-throughput techniques like liquid chromatography–mass spectrometry (LC-MS) and computational tools such as machine learning (ML), its potential for clinical applications has been significantly enhanced. Untargeted metabolomics, typically conducted on high-resolution mass spectrometry (HRMS), is valuable for candidate biomarker screening.^36^ In contrast, targeted metabolomics, often performed using low-resolution mass spectrometry (LRMS), is suitable for routine hospital assays due to its better sensitivity, lower cost, and simpler maintenance.^37,38^ Ensuring the robustness and transferability of biomarker monitoring on different instruments is a crucial prerequisite for the clinical application of metabolomics results.

Herein, metabolomics analysis employing LC-HRMS was conducted on the serum samples obtained from ED-visited patients diagnosed with septic shock or nonseptic shock. A panel of metabolites distinguishing septic shock from nonseptic shock was developed using a generalized linear model (GLM) algorithm. Furthermore, monitoring of this metabolic panel was also established on an LC-LRMS. This study presents a new approach with a short turnover time and high specificity and sensitivity for predicting septic shock occurrence during hospitalization through analysis of serum collected upon admission, thereby assisting physicians in facilitating timely intervention, appropriate triage, and optimal resource utilization.

## Experimental Section

### Study Design and Patient Selection

Adult patients who visited the emergency department of Taipei Veterans General Hospital between Nov. 2018 and Feb. 2019 were prospectively enrolled with approval from the Institutional Review Board (IRB: V109C-012). As illustrated in Figure 1A, a total of 124 patients enrolled were categorized into four subgroups according to the final adjudication: (i) 28 noninfectious controls (ill without any evidence of infection), (ii) 34 nonseptic infection patients (definite infection without progression to sepsis), (iii) 32 nonshock sepsis patients (sepsis without progression to septic shock) and (iv) 32 septic shock patients. These patients were divided into a septic shock group (iv) and a nonseptic shock group (i, ii, iii). The criteria for determining infection status and final adjudication are recorded in the Supporting Information. Serum samples were obtained during routine procedures at the time of ED admission before treatment. Clinical data, including age, gender, temperature, respiratory rate, blood pressure, CRP, PCT, alkaline phosphatase (Alk-P), lactate, white blood cell (WBC), neutrophils, monocytes, and 28-day mortality, were followed-up recorded. There were no differences in temperature, PCT, alkaline phosphatase (Alk-P), and CRP between the two groups, apart from a significantly higher lactate and white blood cell counts (WBC) level in septic shock (Table 1).

**Figure 1:**
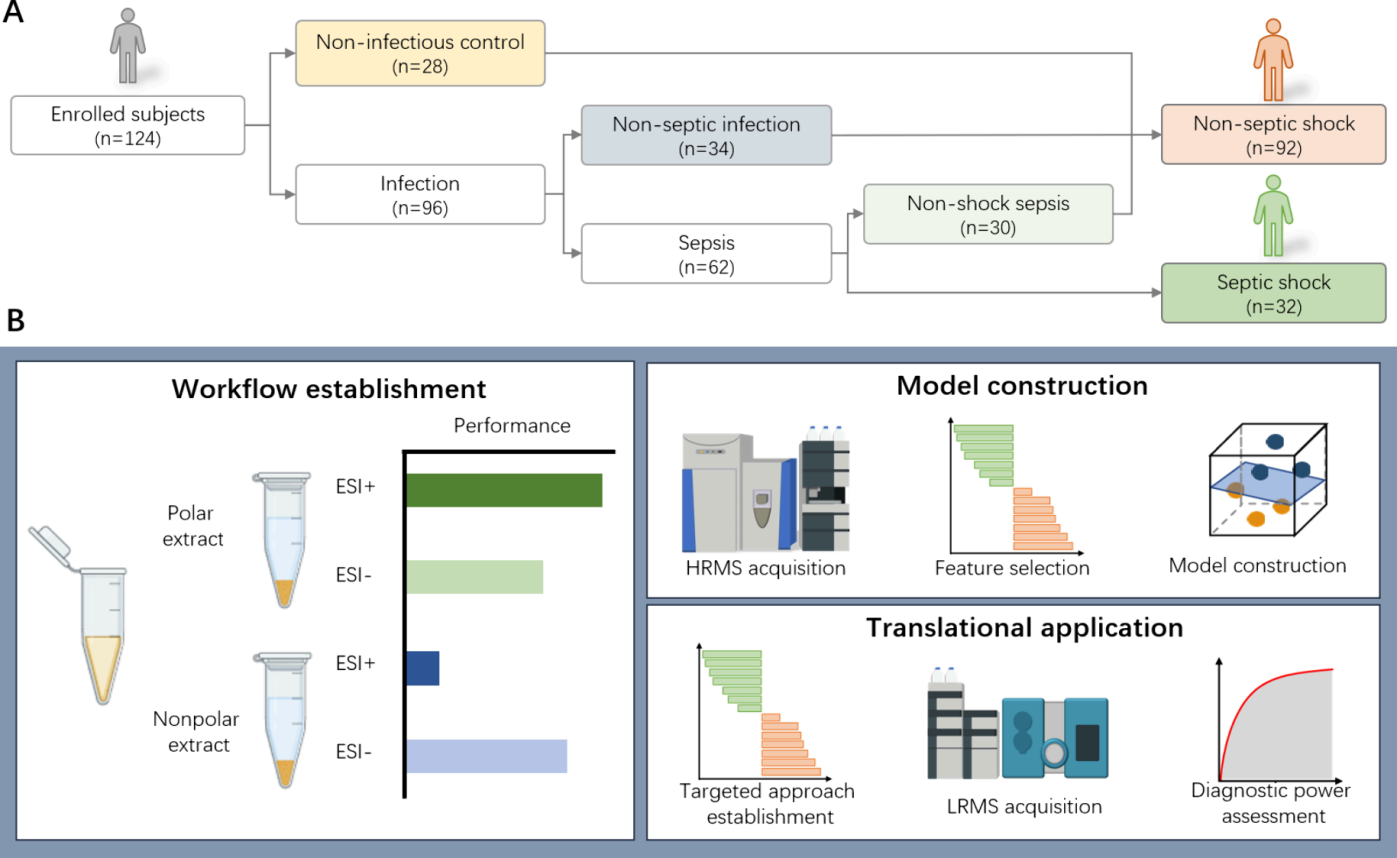
**Overview of study design.** (A) Patient enrollment. (B) Analysis strategy of metabolomics. Performance refers to the classification ability of ESI+ and ESI– methods, measured by AUROC, accuracy, and sensitivity.

**Table 1:** Clinical Characteristics of Enrolled Subjects^a^

Characteristics	Septic Shock	Nonseptic Shock	*p* ^b^
n	32	92	
Age	77.5 ± 11.5	72 ± 15.3	0.035
Gender (% male)	62.5%	53.3%	0.066
Temperature (°C)	37.9 ± 1.6	37.9 ± 1.4	0.863
PCT (ng/mL)	9.3 ± 18.2	3.7 ± 7.2	0.215
Alk-P (U/L)	157.3 ± 87.5	141.6 ± 76.6	0.278
CRP (mg/dL)	14.3 ± 11.1	10.2 ± 10.1	0.052
Lactate (mg/dL)	43.5 ± 27.5	20.1 ± 15	0.000
WBC (10^3^/μL)	16400 ± 11900	10900 ± 5800	0.000
Mortality (%)	37.5%	22.8%	0.912

a Results are expressed as the number (%) or means ± standard deviation. Abbreviations: PCT, procalcitonin; Alk-P, alkaline phosphatase; CRP, C-reaction protein; WBC, white blood cell.

b Septic shock and nonseptic shock patients were compared using Student’s *t* test; proportions were compared using Chi-square.

The study design included three distinct components (Figure 1B). First, a test subset containing 80 samples was utilized to evaluate the metabolomic analysis protocol. The evaluation encompassed different extraction methods and two polarity modes on an LC-HRMS. Subsequently, the untargeted metabolomics method was applied to the entire cohort (n = 124) to acquire the metabolic profiles. An ML workflow was employed to identify a panel of metabolites capable of distinguishing septic shock from nonseptic shock cases for early septic shock prediction model construction. Finally, a targeted detection method for selected metabolites was established on a triple quadrupole MS, the most popular MS instrument in clinical laboratories. Sixty samples were included for targeted detection and evaluation of the prediction performance of the metabolite panel.

### Chemicals

Ultrapure water was prepared using a Milli-Q system (Merck Millipore, Burlington, MA, USA). All solvents and additives used to prepare mobile phases were LC-MS grade. Methanol and butanol were procured from Honeywell (Charlotte, NC, USA), while acetonitrile was sourced from J.T. Baker (Phillipsburg, NJ, USA). Ammonium formate, formic acid, isopropanol, and asymmetric dimethylarginine were acquired from Sigma-Aldrich (St. Louis, MO, USA). 1-methyladenosine, hypaphorine, linoleyl-carnitine, and N-acetylcadaverine were purchased from Toronto Research Chemicals (Toronto, ON, Canada). 15:0–18:1­(d7) phosphatidylcholine were purchased from Avanti Polar Lipids (Alabaster, AL, USA).

### Sample Preparation

Serum samples were extracted using two established single-phase methods for method assessment: methanol for polar metabolites^39^ and 1-butanol/methanol (1:1, v/v) for nonpolar metabolites.^40^ An aliquot of 5 μL serum was extracted with 50 μL of solvent. After vortexing for 10 s, samples were centrifuged for 2 min at 12,000 rpm to precipitate proteins, and the supernatant was collected for mass spectrometry-based metabolomics analysis. For pooled quality control (QC) samples, aliquots from the original samples were pooled together to create a representative QC sample.

### Untargeted Metabolomics Using LC-HRMS

All samples were analyzed utilizing Ultimate 3000 UHPLC coupled with Q Exactive Plus (Thermo Fisher Scientific). For polar extracts, liquid chromatography was performed with an Atlantis HILIC column (3 μm, 2.1 mm × 100 mm, Waters) at 30 °C. Nonpolar extracts were separated on a BEH C18 column (1.7 μm, 2.1 mm × 100 mm, Waters) at 40 °C. The exact gradient conditions can be found in the Supporting Information. The MS with an electrospray ionization (ESI) source was operated separately in positive (ESI+) and negative (ESI−) modes. Operating conditions included a source temperature of 200 °C, capillary temperature of 250 °C, and spray voltage of 3.5 kV. Full-scan MS spectra (*m*/*z* 70–1500) were acquired at a resolution of 60,000. The top ten most intense ions were selected with an isolation window of 2 Da for MSn fragmentation (20–40% stepped normalized collision energy). Raw data files were processed using the Compound Discoverer 2.1 (CD, Thermo Fisher Scientific) built-in workflow, including peak alignment, detection, and integration. Detailed parameters of the workflow were provided in the Supporting Information. Briefly, compounds with an intensity greater than 100,000 and a signal-to-noise ratio greater than ten were extracted from the files. They are then combined into groups based on a mass tolerance of less than 5 ppm and a retention time shift of less than 0.5 min. A procedural blank sample was used for background subtraction and noise removal. Peak areas across all samples were normalized using the pooled quality control (QC) samples to correct for variations resulting from instrument instability. Finally, chromatographic peaks with the exact *m*/*z* × RT dimensions were extracted as metabolic features.

### Targeted Measurement Using LC-LRMS

Targeted measurements were conducted on an LC-LRMS system (5500+ Triple Quad system, SCIEX) in positive ionization mode (ESI+). Chromatographic separation for targeted analysis was performed using the HILIC method that was identical in the untargeted analysis. Source parameters were as follows: ion spray voltage, 5500 V; source temperature, 550 °C; ion source gas, 55 psi; curtain gas, 20 psi. Targeted compound detection utilized multiple reaction monitoring (MRM). MRM transitions were first tuned using commercial internal standards. For metabolites lacking standards or annotations, transitions were determined by analyzing the serum mixture sample at various CE, DP, EP, and CXP settings in parallel LC-MS runs. For semiquantitative determination, the internal standard 15:0–18:1­(d7) phosphatidylcholine was spiked into each sample at a final concentration of 2 ppm to normalize the signal intensity. Data analysis was performed using Analyst software (SCIEX).

### Metabolite Annotation

Metabolites were identified by comparing exact mass (MS1), RT or fragmentation pattern (MS/MS) to authenticated standards, extensive molecular structural databases (mzCloud, Human Metabolome Database,^41^ Metlin,^42^ and PubChem^43^), or in-silico software (SIRIUS^44^). The confidence levels of metabolite annotations were defined by Metabolomics Standards Initiative Chemical Analysis Working Group report^45^ as follows: level 1 for metabolites confirmed using authentic metabolites standards with three orthogonal properties (i.e., MS1 + RT + MS/MS); level 2 for metabolites putatively confirmed by library-based annotations using two orthogonal properties (i.e., MS1 + MS/MS); level 3 for chemical class prediction using CANOPUS^46^ from Sirius^44^ according to Classyfire taxonomy;^47^ level 4 for unknown compounds. The MS/MS cosine similarity was calculated using filtered spectra (the top 15 most intense ions were included) to measure how well the spectra matched.

### Model Construction and Statistical Analysis

The prediction model was constructed using a generalized linear model (GLM) in a commercially available software, RapidMiner (version 9.2.001). The data set was randomly divided into a training set (70%) and a test set (30%), ensuring consistent class proportions. A two-step feature selection was performed on the training set. First, in the frequency-based selection, 20 iterations were performed. The model was trained on a randomly selected 90% subset of the training data in each iteration, assigning weights to metabolic features. Features that were consistently assigned weights in at least ten out of the 20 iterations (frequency ≥ 50%) were retained. Second, in the backward sequential selection, the least contributing features were removed one by one until a subset that maximized the model’s performance was achieved. The selected features were used to train a model on the entire training set, and performance was evaluated using leave-one-out cross-validation. Finally, the model was assessed using the test set.

The descriptive statistics for models encompass accuracy, sensitivity, specificity, and area under the receiver operating characteristic curves (AUROC). The unsupervised principal component analysis (PCA) was conducted using Origin Pro 9.0. Receiver operating characteristic curves (ROC) analysis and violin plot were employed using GraphPad Prism 6. Differences between groups were assessed through a two-tailed Student’s *t* test or the Chi-square test as appropriate.

## Results

### Optimizing Metabolomics Protocols for Septic Shock Prediction

To conserve research resources, 80 serum samples, including 20 from septic shock and 60 from nonseptic shock, were selected as a test cohort to evaluate the untargeted analysis protocol. The nonseptic shock group comprised 20 samples from each disease subgroup: noninfectious controls, nonseptic infection, and nonshock sepsis. Four metabolomics methods were conducted using an LC-HRMS to profile the global serum metabolites of this cohort. These methods included two extraction procedures with varying polarity coverage:^39,40^ “polar” and “nonpolar”. Each was paired with positive (ESI+) and negative (ESI−) ionization modes. Representative total ion current chromatograms (TIC) of these methods (polar ESI+, polar ESI–, nonpolar ESI+, and nonpolar ESI−) were shown in septic shock and noninfectious control, as shown in Figure S1. As demonstrated by the PCA (Figure 2A), we found no general differences between the septic shock group and the nonseptic shock group under all the four acquisitions. The QC samples were clustered tightly in unsupervised PCA model, demonstrating the stability and feasibility of the four acquisition methods.

**Figure 2:**
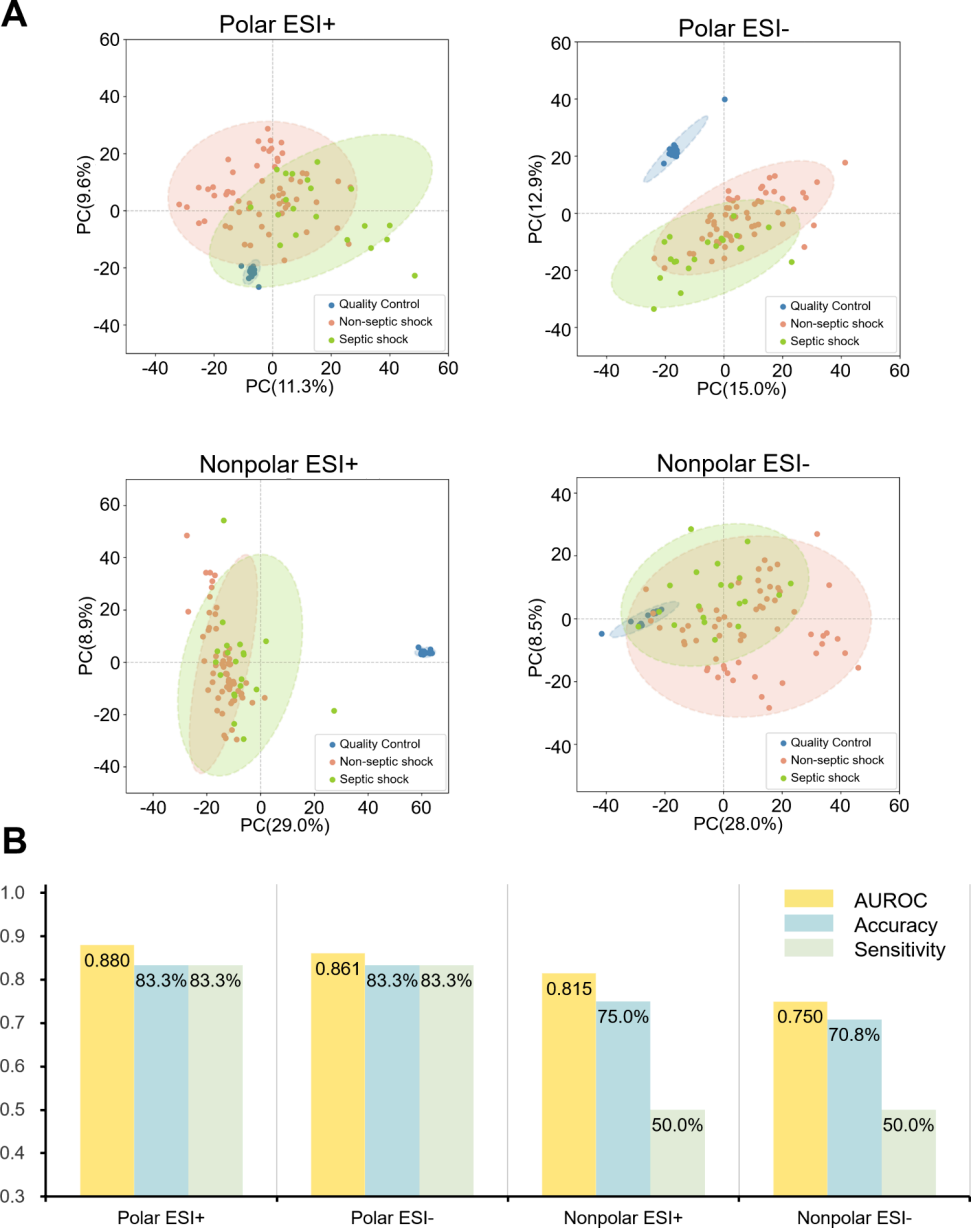
**Method evaluation.** PCA score plot (A) and the classification performance (B) of the 4 acquisition methods, including polar ESI+, polar ESI–, nonpolar ESI+, and nonpolar ESI–.

Given PCA’s limitations in discerning significant differences, supervised GLM was employed in each data set for septic shock prediction. The data set was randomly divided into a training and a test set. The train-test splits were consistent across the four data sets for uniform evaluation. The classification performances, represented by the AUROC, accuracy, and sensitivity, were summarized in Figure 2B. Generally, the classification performance of polar methods was better than that of nonpolar methods, with higher AUROC and accuracy in both ESI+ and ESI– acquisition modes. Among the models based on polar extraction, the ESI+ method demonstrated the highest performance in discriminating septic shock (AUROC = 0.880).

While wider coverage of metabolite detection can be beneficial for identifying disease markers, clinical application requires consideration of complexity and time efficiency. Minimizing operational steps and costs is crucial for practical implementation. Therefore, despite the broader metabolite coverage achievable with multiple methods, we selected the polar extraction method combined with ESI+ HRMS acquisition as the optimal metabolomics pipeline for subsequent analyses. This optimal method was then applied to a larger cohort to develop and validate a predictive model for septic shock, as detailed in the next section.

### Development and Validation of a Metabolomics-Based Predictive Model for Septic Shock

Building on the optimized acquisition identified in the previous section, we performed the untargeted metabolomics analysis on a complete cohort, including 32 patients with septic shock and 92 patients with nonseptic shock. A total of 2892 metabolic ion features were observed in the serum samples. Consistent with previous PCA plot results, minimal differences between septic shock and nonseptic shock were observed, with QC samples tightly clustered (Figure S2A).

An ML pipeline workflow (Figure 3A) was applied to identify the optimal combination of metabolites for septic shock classification. The data set was randomly divided into a training set for feature selection and model construction, and a test set for evaluation. Initially, in the frequency-based ranking selection, 55 features that were weighted at least ten times out of 20 modeling iterations were retained as the initial candidate subset (Figure 3B). Subsequently, we used greedy backward elimination to simplify the model by iteratively eliminating the least contributing features from the candidate subset. As illustrated in the relationship between model performance and the number of features (Figure 3C), accuracy and sensitivity increased as the number of chosen metabolite features was reduced from 55 to 22. Furthermore, compared to the original 2892 feature data, the PCA score plot of the 22 discriminant features exhibited improved clustering (Figure S2B).

**Figure 3:**
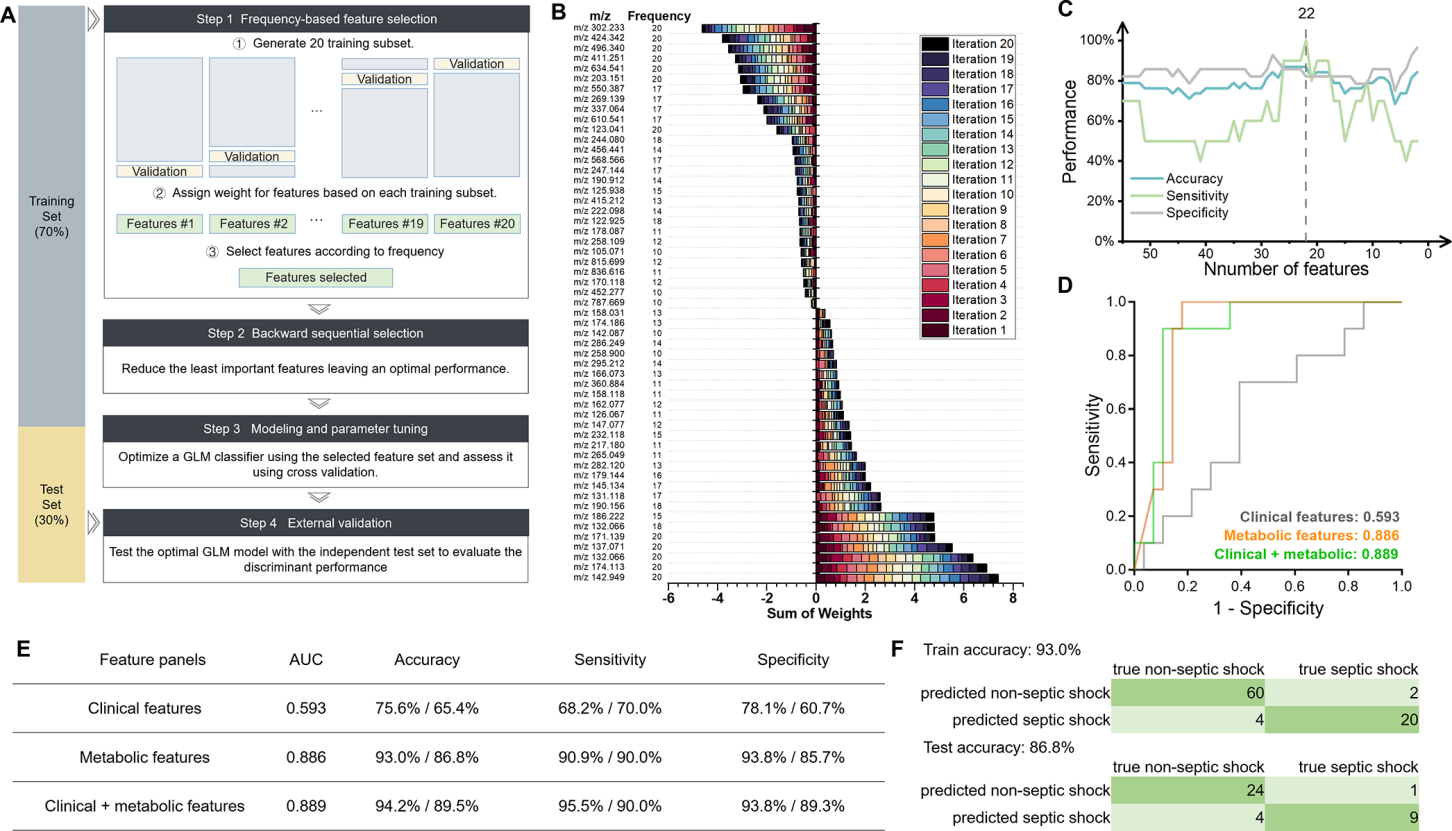
**Model construction.** (A) Machine learning pipeline. (B) The sum of feature weights by frequency-based feature selection (step 1). (C) Performances for each feature size eliminated by sequential backward selection (step 2). ROC curve (D) and performance summary (E) of 3 septic shock classifiers using the clinical feature panel, metabolic feature panel, and their combined panel. Performance is shown as training/test values. (F) Confusion matrix of the metabolic-features-based model in the training set (up) and the test set (down).

The 22 metabolic features, characterized by fewer features but superior performance, were selected as a panel for septic shock prediction. For comparison, clinical features collected upon admission, including traditional sepsis biomarkers like CRP, PCT, lactate, and WBC, were also used for modeling. Cross-validation was performed on the training set, and the model was validated using the test set. The classifier’s effectiveness was represented by the ROC curve for the test set (Figure 3D), with other classification performance metrics summarized in Figure 3E. The metabolite-based model outperformed the clinical feature-based model across all metrics, achieving an AUROC of 0.886, an accuracy of 86.8%, a sensitivity of 90.0%, and a specificity of 85.7%. In contrast, the clinical feature-based model yielded an AUROC of 0.593, with lower accuracy (65.4%), sensitivity (70.0%), and specificity (60.7%). The confusion matrix further highlights the superior performance of the metabolite-based model (Figure 3F). In the training set (n = 86), 80 subjects were classified correctly, resulting in an average accuracy of 93.0%. In the test set (n = 38), 33 subjects were classified correctly, resulting in an accuracy of 86.8%. Integrating the metabolic feature panel with the clinical features further enhanced prediction performance, achieving 89.5% accuracy and an AUROC of 0.889 (Figure 3E). However, the improvement in septic shock prediction by combining clinical data was not substantial. Additionally, collecting these multidimensional clinical data in the ED takes considerable time, particularly for laboratory test results. Therefore, while integrating clinical data can enhance predictive capability, its cost-effectiveness may be limited due to the response time constraints in acute settings.

### Targeted Metabolite Detection for Clinical Application

Using the multiple reaction monitoring (MRM) mode on a triple quadrupole LRMS is a widely used strategy in clinical routine due to its economic and technical advantages.^37,38^ Demonstrating the robustness and transferability of the platform is essential for practical applications. Therefore, we transferred the acquisition method from an LC-HRMS to an LC-LRMS to demonstrate the feasibility of targeted detection of selected metabolites for predicting septic shock. We evaluated the targeted platform using a cohort of 62 patients, including 32 with septic shock and 30 without. A total of 42 transitions were included in a targeted MRM detection for 22 metabolites (Table S1). This method was implemented on an LC-LRMS to detect key serum metabolites in the cohort.

The ROC curve for septic shock prediction in the test data set (Figure 4A) showed an AUROC of 0.811, significantly outperforming the clinical model. The effectiveness of the metabolite-based model is further illustrated by the confusion matrix (Figure 4A), with prediction accuracies exceeding 80% in both the training and test sets. Specifically, in the training set, the model correctly predicted 18 out of 22 septic shock cases (sensitivity = 81.82%), and in the test set, it correctly predicted 9 out of 10 cases (sensitivity = 90.0%). The LRMS-based model maintained its sensitivity, accuracy, and AUROC compared to the HRMS-based model, underscoring the robustness of the prediction platform based on 22 metabolic features, irrespective of instrument resolution. This demonstrates the feasibility and efficiency of targeted metabolite detection in routine clinical practice. The high sensitivity and accuracy across different platforms confirm that the panel is reliable for septic shock prediction, ensuring the method can be integrated into clinical workflows without compromising diagnostic performance.

**Figure 4:**
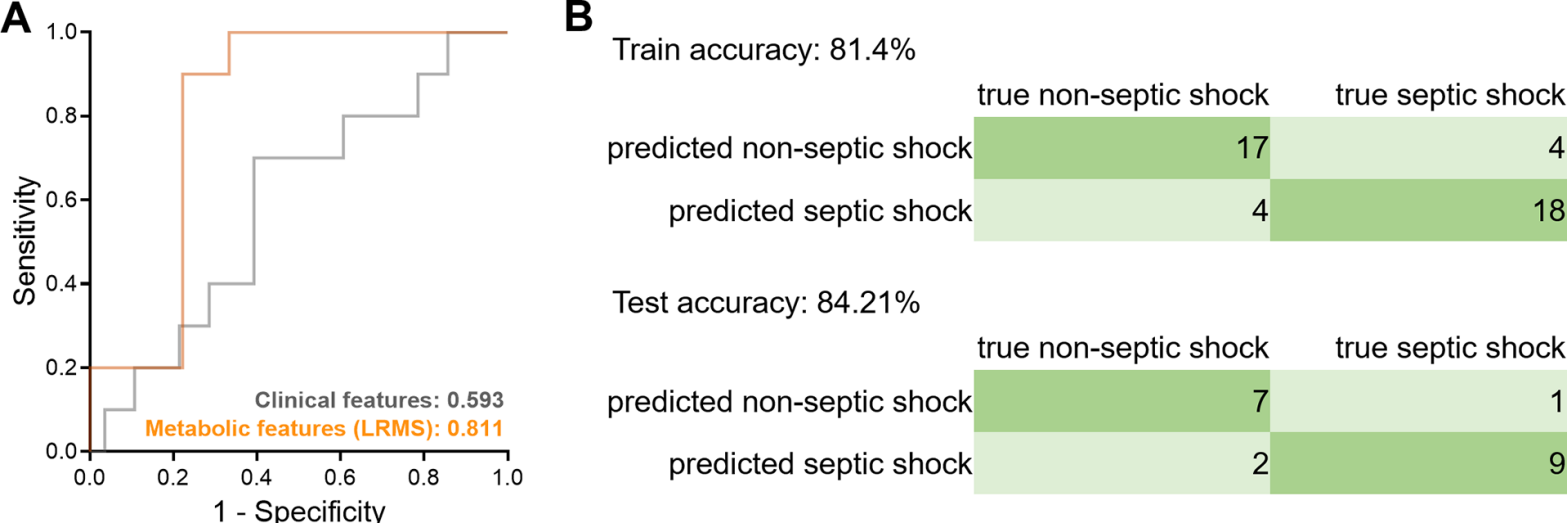
**Performance of the targeted method**. (A) ROC curve of septic shock classifier using the metabolic features measured by LRMS. The ROC of the clinical panel is also displayed in the diagram for comparison. (B) Confusion matrix in the training set (up) and the test set (down) of the model constructed on metabolic features detected by LRMS.

### Identification of Metabolic Markers and Their Clinical Significance in Septic Shock

Among the metabolites in the discriminant feature panel, nine exhibited positive weights, and 13 exhibited negative weights, contributing to the prediction of septic shock (Figure 5A). The metabolic features were further annotated according to the guidelines proposed by the Metabolomics Standards Initiative,^45^ levels 1 for authentic standards-based annotation, levels 2 for library-based annotations, and level 3 for chemical class assignment using in-silico tools (Table 2, Figures S3–12). As a result, ten of the metabolites were unequivocally identified (Table 2), including 1-methyladenosine, N-acetylcadaverine, trigonelline, 5-amino-levulinic acid, linoleyl-carnitine, hypaphorine, phosphatidylcholine (20:1/0:0) (PC (20:1/0:0)), dihydroceramide (d18:0/18:0) (DHCer (d18:0/18:0)), asymmetric dimethylarginine/symmetric dimethylarginine (ADMA/SDMA), and N-acetyl-d-galactosamine. Given the structural similarity of these isomers and the absence of clear chromatographic separation in our current method, ADMA and SDMA are discussed together in this study. Univariate ROC curve analysis showed that the AUROC of individual metabolites was less than 0.8, except for N-Acetylcadaverine, indicating that a single metabolite cannot effectively discriminate septic shock patients from nonseptic shock. This result emphasizes the advantage of multivariate analysis, as combining multiple metabolites offers better discriminatory power than using individual biomarkers—a concept well supported in the literature.^25^

**Table 2:** Identification of Metabolites

ID	*m*/*z*	RT	Ion	Molecular Formula	Compound Class	Compound Identification	Level^b^	*p* [Table-fn t2fn3]	AUC
2018	156.102	1.4	[M + H]^+^	C_8_H_13_NO_2_	Amino acids and derivatives		3	8.4 × 10^–3^	0.69
1567	132.066	1.5	[M + H]^+^	C_5_H_9_NO_3_	Amino acids and derivatives	5-aminolevulinic acid	2	2.2 × 10^–2^	0.65
2202	132.066	1.8	[M + H]^+^	C_5_H_9_NO_3_	Amino acids and derivatives		3	5.0 × 10^–4^	0.70
2073	137.071	5.6	[M]^+^	C_7_H_9_N_2_O^+^	Pyridinecarboxamides	trigonellinamide	2	1.7 × 10^–2^	0.68
1519	174.186	2.7	[M + H]^+^	C_10_H_23_NO	Alkanolamines		3	7.0 × 10^–1^	0.59
2848	282.12	5.0	[M + H]^+^	C_11_H_15_N_5_O_4_	Purine nucleosides	1-methyladenosine	1	1.0 × 10^–4^	0.79
2074	145.134	5.1	[M + H]^+^	C_7_H_16_N_2_O	Carboxylic acid amides	N-acetylcadaverine	1	6.5 × 10^–3^	0.69
1965	415.212	1.1	[M + H]^+^	C_24_H_30_O_6_	Phenol ethers		3	6.2 × 10^–2^	0.61
1602	244.08	2.1	[M + Na]^+^	C_8_H_15_NO_6_	Monosaccharides		3	4.7 × 10^–2^	0.63
2037	247.144	4.5	[M + H]^+^	C_14_H_18_N_2_O_2_	Amino acids and derivatives	hypaphorine	1	1.7 × 10^–2^	0.69
2535	105.071	1.1	[M – H_2_O + H]^+^	C_8_H_10_O	Cresols		3	3.0 × 10^–3^	0.70
2041	222.098	2.2	[M + H]^+^	C_8_H_15_NO_6_	Monosaccharides	N-acetyl-d-galactosamine	2	6.3 × 10^–1^	0.53
1712	190.912	4.8	-	-	-	-	4	1.0 × 10^–4^	0.73
2343	568.566	1.7	[M + H]^+^	C_36_H_73_NO_3_	Ceramides	DHCer (d18:0/18:0)	1	1.1 × 10^–2^	0.65
84	125.938	5.1	-	-	-	-	4	1.0 × 10^–4^	0.75
2485	178.087	7.2	[M + H]^+^	C_10_H_11_NO_2_	Amino acids and derivatives		3	6.6 × 10^–3^	0.68
124	456.441	1.8	[M + H]^+^	C_28_H_57_NO_3_	N-acyl amines		3	4.6 × 10^–2^	0.59
185	550.387	5.3	[M + H]^+^	C_28_H_56_NO_7_P	Glycerophosphocholines	PC (20:1/0:0)	2	1.0 × 10^–4^	0.75
2617	424.342	4.1	[M + H]^+^	C_25_H_45_NO_4_	Acyl carnitines	linoleyl-carnitine	1	5.0 × 10^–4^	0.76
374	269.139	1.2	[M + H]^+^	C_14_H_20_O_5_	Carboxylic acid esters		3	2.3 × 10^–2^	0.65
298	302.233	1.1	[M + H]^+^	C_16_H_31_NO_4_	Amino acids and derivatives		3	6.0 × 10^–4^	0.73
2115	203.151	7.9	[M + H]^+^	C_8_H_18_N_4_O_2_	Amino acids and derivatives	ADMA/SDMA	1	9.0 × 10^–1^	0.60

a Level was the annotation confidence.

b The *p* value was acquired for septic shock and nonshock through Student’s *t* test.

**Figure 5:**
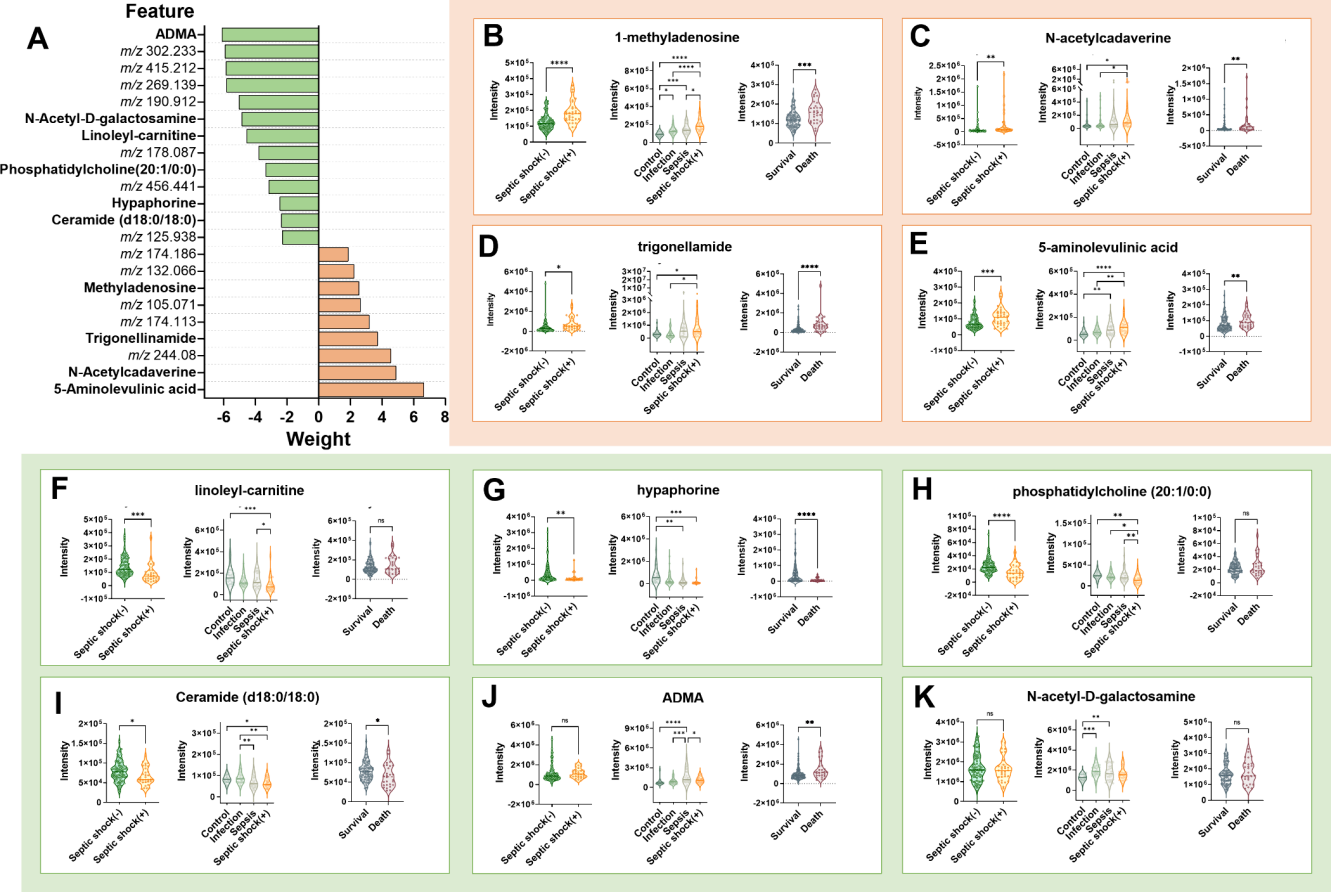
**Multivariate statistical analysis of the metabolic panel.** (A) The weight of the 22 metabolic features in the model. Serum metabolite levels between septic shock and nonseptic shock, between different disease severities (control, infection, sepsis, and septic shock), and between death and survival groups, including four metabolites with a positive weight (B to E) and six with a negative weight (F to K). *P < 0.05, **P < 0.01, ***P < 0.001, and ****P < 0.0001.

Significant changes in serum level were observed in 18 metabolites between the septic shock and the nonseptic shock with a two-sample student *t* test (Table 2, Figure S13). Patients with septic shock had higher serum concentrations of 5-aminolevulinic acid, trigonelline, 1-methyladenosine, N-acetylcadaverine, and two amino acids and derivatives (ID-2018_*m*/*z* 156.102; ID-2202_*m*/*z* 132.066) compared to nonseptic shock patients (Figure 5B–E, Figure S13). Significant downregulation of 12 metabolites was observed in septic shock, including hypaphorine, DHCer (d18:0/18:0), PC (20:1/0:0), linoleyl-carnitine, one monosaccharide (ID-1602_*m*/*z* 244.080), one cresol (ID-2535_ *m*/*z* 105.071), one N-acyl amine (ID-124_*m*/*z* 456.441), one carboxylic acid ester (ID-374_*m*/*z* 269.139), one amino acid and derivative (ID-298_*m*/*z* 302.233) and two unknown compounds (ID-1712_*m*/*z* 190.912; ID-84_*m*/*z* 125.938) (Figure 5F–I, Figure S13). Notably, ADMA/SDMA, N-acetyl-d-galactosamine, one alkanolaminesand (ID-1519_*m*/*z* 174.186), and one phenol ether (ID-1965_*m*/*z* 415.212) did not differ statistically between septic shock and nonseptic shock patients (Figure 5J,K, Figure S13).

The results from multiple *t* tests indicated consistent trends in metabolite changes associated with increasing severity, from noninfection control to septic shock, and correlated with 28-day survival outcomes (Figures S13–15). Nonseptic shock patients were categorized into three groups based on the severity of their condition: control (denotes noninfection control), infection (denotes nonsepsis infection), and sepsis (denotes nonshock sepsis). Among the identified metabolites, 1-methyladenosine accumulated significantly in septic shock compared to control (*p* < 0.0001), infection (*p* < 0.0001) and sepsis (*p* < 0.05) (Figure 5B). It also increased in sepsis compared to control (*p* < 0.001) and in infection compared to control (*p* < 0.05), indicating a severity-dependent elevation. 5-aminolevulinic acid also showed increased levels in septic shock group (vs control, *p* < 0.0001; vs infection, *p* < 0.01), and in sepsis group (vs control, *p* < 0.01) (Figure 5E). PC (20:1/0:0) demonstrated remarkable depletion in septic shock (vs control, *p* < 0.01; vs infection, *p* < 0.05; vs sepsis *p* < 0.01) (Figure 5H). Additionally, patients were divided into survival and death groups based on the results of the 28-day follow-up. Seven metabolites were associated with poor patient survival, including 1-methyladenosine (Figure 5B), N-acetylcadaverine (Figure 5C), trigonelline (Figure 5D), 5-amino-levulinic acid (Figure 5E), hypaphorine Figure 5G), DHCer (d18:0/18:0) (Figure 5I) and ADMA/SDMA (Figure 5J). These observations suggest probable associations between these metabolites and disease progression and outcome.

## Discussion

This study integrated metabolomics and machine-learning techniques to develop an early prediction platform for septic shock within an ED setting, where inadequate monitoring and care resources necessitate rapid recognition and triage. Distinguished from studies focusing on ICU populations or high-risk cohorts, our research collected sera upon admission from a diverse cohort reflecting routine ED practice, capturing a spectrum of severity from mild noninfectious diseases to critical septic shock. The clinical implications of this study are multiple. First, we demonstrated that an algorithm utilizing several useful metabolites can provide excellent predictive characteristics for identifying patients at high risk of septic shock. We then showed the retentive favorable predictive ability of the metabolite panel measured using another clinically practical mass spectrometer, improving its generality and transferability. Finally, statistical correlations of these metabolites with disease progression enable insights into their biological roles, addressing the critical need for timely recognition in early admission stages.

Based on these results, we propose a strategy for early recognition of septic shock using serum metabolic classifiers, as shown in Figure 6. In this strategy, sample collection is performed immediately after patient admission. The follow-up analysis includes metabolite extraction and LC-MS analysis, with a turnaround time of around 30 min. Typically, the diagnostic process involves monitoring the patient’s condition using the SOFA scoring system, which requires periodic data measurements to be completed, making it time-consuming and impractical in the ED. In contrast, our strategy leverages LC-MS technology to achieve high throughput measurements of all biomarkers with minimal operations. This approach offers a short handling time and enables early risk assessment of septic shock, facilitating timely intervention and subsequent disease management.

**Figure 6:**
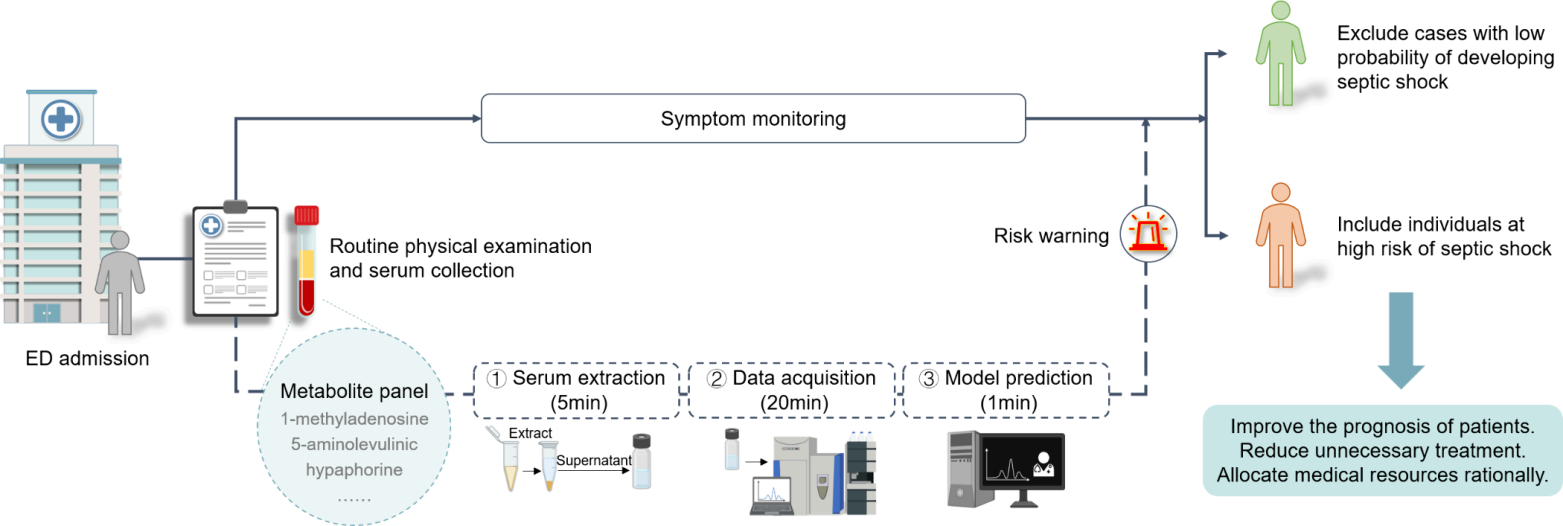
**Proposed classification strategy for septic shock.** A typical diagnostic process involves monitoring patient condition using the SOFA scoring system, which requires periodic data measurements to be completed, making it time-consuming and impractical in the ED. Our platform utilizes serum metabolic classifiers with diagnostic value, which offer short handling time and early risk assessment of septic shock, facilitating timely intervention and subsequent disease management.

Previous efforts to predict septic shock largely depended on electronic health records (EHRs), which provide extensive longitudinal data, including demographics, laboratory results, and vital signs, to monitor patient health over time. Wardi et al. developed an ML-based system recognizing delayed septic shock onset in an 8–12 h window using EHR variables.^28^ Kim et al. combined traditional biomarkers with EHR to identify septic shock within 24 h, outperforming the qSOFA score and MEWS score.^48^ While machine-learning-based classifiers using EHR data offer significant advantages in terms of big data utilization for disease diagnosis, there are inherent limitations. The quality and consistency of EHR data can vary widely across different healthcare systems, which may affect the reliability of predictive models. Additionally, the variable intervals at which data is collected can pose challenges in accurately determining the temporal relationships in clinical data, potentially limiting the generalizability of these diagnostic tools.

Current novel diagnosis tools primarily focus on sepsis detection rather than specifically targeting septic shock, as well as their time efficiency often falls short of the urgent demands of emergency settings. Techniques, such as PCR and Fluorescent In-Situ Hybridization can detect bacterial sepsis rapidly, sometimes within 30 min.^49^ However, these methods often rely on positive blood cultures, which require additional time for bacterial growth before measurement. Although several blood plasma assays and leukocyte technologies show promise, achieving both the required accuracy and speed within an hour has proven challenging.^49^ Our approach, by directly measuring metabolites in blood samples, provides a more consistent and rapid means of detection.

Metabolomics has shown potential in sepsis diagnosis, yet its application in predicting septic shock, particularly in the ED setting, remains relatively unexplored. For example, Kauppi et al. constructed a six-metabolite predictive model for bacteremic sepsis, outperforming conventional biomarkers like CRP and WBC.^50^ Similarly, Langley et al. investigated the plasma metabolome and the clinical features of patients upon ED arrival.^51^ The algorithm, formed from five metabolic features and two clinical features, demonstrated superior predictive value for sepsis outcomes compared to a traditional marker or the SOFA score. However, to the best of our knowledge, rare studies have utilized metabolomics to predict septic shock in the ED.

Our strategy integrates metabolomic analysis with ML techniques, offering significant benefits for predicting septic shock in the ED. We have transitioned measurements from LC-HRMS to LC-LRMS, and both models built on these measurements consistently demonstrated excellent performance, highlighting the effectiveness and feasibility of our approach. Although untargeted HRMS methods have been valuable in discovery research due to their high resolving power, targeted LRMS methods provide notable advantages such as reduced setup costs and easier maintenance, making them particularly suitable for routine clinical use.^37,38^ Consequently, we employed HRMS for biomarkers discovery while adopting LRMS to develop practical methods that can accommodate laboratories with diverse instrument setups. LC-MS techniques allow for high-throughput processing with minimal operational requirements, and the typical turnaround time for each sample in our strategy is approximately 30 min, encompassing both sample extraction and LC-MS analysis. This workflow can be further streamlined through automation, potentially reducing manual operations and enhancing efficiency. Immediate sampling upon ED admission also aims to minimize the influence of treatment on metabolites and supporting early disease prediction.

In this study, a panel of metabolites was selected for septic shock discrimination. Examining the metabolites’ biological role enables interpretation of their potential biological roles in the disease progression of septic shock. Elevated levels of 5-aminolevulinic acid, the first intermediate in the heme biosynthetic pathway catalyzed by 5-aminolevulinic acid synthase (ALAS), suggest an activation of this pathway during septic shock. This aligns with previous studies that have noted increased heme biosynthesis in a high-risk group of sepsis,^52^ where upregulation of the *ALAS2* (ALAS enzyme-coding gene) has been observed to mitigate tissue damage.^53^ While this suggests a possible role of 5-aminolevulinic acid in the progression of septic shock, further investigation is needed to confirm its specific contributions.

Alterations in nucleoside metabolism are also evident in septic shock. Increased 1-methyladenosine levels may suggest heightened RNA turnover in response to inflammatory stress, a marker that has been linked to poor outcomes in severe infections^33^ and other inflammatory conditions.^54–56^ This modified nucleoside, mainly found in transfer and rRNAs, is released in response to oxidative DNA damage and systemic RNA turnover,^57^ marking it as a potential indicator of oxidative stress and inflammation.

Other metabolites suggest significant microbial activity and immune response disruptions. N-acetylcadaverine is acetylated from cadaverine, which is not biosynthesized in humans but is available in bacteria.^58^ Its accumulation points to bacterial activity and infection, consistent with the role of polyamines in immune response and pathogen-induced inflammation.^59,60^ Trigonelline (1-methylnicotinamide), produced by NAD metabolism in the liver, was significantly increased in patients with septic shock and in those who did not survive. Abnormal NAD metabolism is common in aging and infection,^61–63^ where high expression of nicotinamide *N*-methyltransferase (NNMT) converts nicotinamide to 1-methylnicotinamide,^63^ removing excess nicotinamide under inflammatory conditions and affecting global NAD+ levels.^61^

In addition to immune and inflammatory responses, alterations in nitric oxide (NO) metabolism also play a crucial role. ADMA and SDMA, two structurally related methylated derivatives of arginine, have been extensively studied in the context of vascular dysfunction and sepsis.^64,65^ ADMA is a well-known endogenous inhibitor of NO synthase, while SDMA affects NO availability by competing with arginine for cellular transport. Dysregulated NO production has been implicated in endothelial dysfunction, vasodilation abnormalities, and multiorgan failure in sepsis.^65,66^ In our study, ADMA/SDMA levels showed a slight increase in septic shock patients, although the difference was not statistically significant compared to the nonseptic shock group. However, sepsis patients exhibited significantly higher levels compared to infection-only and negative controls, suggesting a potential association with disease severity. These findings align with previous research, where elevated ADMA and SDMA levels correlated with increased illness severity and higher mortality in sepsis patients.^65^

Disruptions in energy and lipid metabolism are also apparent. Reduced hypaphorine levels in septic shock patients, an exogenous metabolite from fungi and plants,^67,68^ suggest potential disruptions in dietary intake or absorption, though direct associations with metabolic diseases remain unexplored. Decreased linoleyl-carnitine levels are indicative of disruptions in fatty acid metabolism, which has been noted in several metabolic disorders due to altered energy metabolism.^69,70^ The significant reduction in lipid markers such as lysophosphatidylcholine (PC (20:1/0:0)) and dihydroceramide (DHCer (18:0/18:0)) highlights the lipid abnormalities that occur in septic shock. Consistent with our result, PC (20:1/0:0) levels have been reported to decrease in sepsis cases and correlate with the severity of sepsis.^71^ Additionally, the inverse association observed with DHCer (18:0/18:0) suggests these dihydroceramides, despite being inert precursors, may serve as biomarkers of metabolic dysfunction, indicating distinct biological functions from more prevalent ceramides. Similar to our finding, a significant reduction in specific dihydroceramide species was reported in patients with septic shock, compared to both sepsis and healthy control.^72^ The relationship between global serum levels of dihydroceramide and septic conditions warrants further investigation.

While our study has indeed provided valuable insights into the potential of metabolic profiling for predicting septic shock, some limitations should be acknowledged. A key consideration is the study population and generalizability. Expanding the study to include diverse populations across multiple healthcare settings, along with prospective validation in a blinded, independent cohort, will be crucial for confirming model robustness before clinical deployment Another important factor is analytical reliability. The semiquantitative metabolomic analysis relied on a single internal standard, which, while useful for correcting technical variations, may not fully account for differences in extraction and ionization efficiencies across metabolites. Future studies should consider using multiple internal standards tailored to different metabolite classes and further evaluate extraction reproducibility and instrument stability to enhance quantification accuracy. In addition, real-world clinical implementation requires further investigation. Since patient samples are processed individually rather than in batches, future studies should assess whether measurement variability remains consistent in single-sample processing compared to batch-based workflows, ensuring diagnostic feasibility. Beyond these methodological refinements, identifying the unknown metabolites reported in this study will be essential for enhancing the biological interpretability of our findings and advancing the broader application of metabolomics in septic shock research.

## Conclusion

In conclusion, our study presents a novel approach for the early prediction of septic shock in the ED by integrating metabolomics and machine learning techniques. By focusing on a diverse cohort reflective of routine ED practice, we have demonstrated that a metabolite-based algorithm can accurately identify patients at high risk for septic shock. The findings contribute to our understanding of the biological underpinnings of septic shock and offer a scalable solution applicable in EDs and diverse healthcare settings. Based on these results, we propose a strategy to optimize risk assessment and streamline clinical management processes, thereby facilitating timely intervention, expediting the triage of septic shock patients, and enhancing the efficient allocation of medical resources.

## Supplementary Material









## Data Availability

The data used during the current study are available at 10.21228/M84Q71.

## Notes

Detailed experimental parameters and additional results, including information on clinical adjudication, serum collection, the gradient program used for chromatographic separations, MS parameter settings, compound annotations, and preprocessed mass spectrometry data; also included: Figure S1. Representative total ion current chromatograms of the 4 acquisition methods; Figure S2. PCA score plot; Figure S3–12. MSMS fragmentation and LC chromatography of identified compounds; Figure S13. Serum levels of 22 metabolites between septic shock and nonseptic shock; Figure S14. Serum levels of 22 metabolites between different disease severities; Figure S15. Serum levels of 22 metabolites between death and survival groups; Table S1. MRM transitions of 22 metabolites (PDF)
Clinical features (XLSX)
Preprocessed MS data by HRMS (XLSX)
Preprocessed MS data by LRMS (XLSX)
