# Supplementary material for: Early Prediction of Septic Shock in Emergency Department Using Serum Metabolites
Source: J Am Soc Mass Spectrom. 2025 May 9;36(6):1264–76. doi: 10.1021/jasms.5c00009 (PMC12142664; doi:10.1021/jasms.5c00009)
Supplement: Supplementary file 1 [file js5c00009_si_001.pdf]

Supporting Information

Early Prediction of Septic Shock in Emergency Department Using Serum Metabolites

Yu Hong[1,†], Li-Hua Li[2,3,†], Ting-Hao Kuo[1], Yi-Tzu Lee[4,5,*], and Cheng-Chih Hsu[1,6,*]

1 Department of Chemistry, National Taiwan University, 10617, Taipei, Taiwan;

2 Department of Pathology and Laboratory Medicine, Taipei Veterans General Hospital, 11217, Taipei, Taiwan;

3 Ph.D. Program of Medical Biotechnology, Taipei Medical University, 110301, Taipei, Taiwan;

4 European Molecular Biology Laboratory, 69117, Heidelberg, Baden-Württemberg, Germany

5 Department of Emergency Medicine, Taipei Veterans General Hospital, 112201, Taipei, Taiwan

6 Faculty of Medicine, School of Medicine, National Yang Ming Chiao Tung University , 112304, Taipei, Taiwan

7 Leeuwenhoek Laboratories Co. Ltd, 106070, Taipei, Taiwan

† These authors contributed equally to this work.

* Corresponding authors: Cheng-Chih Hsu (ccrhsu@ntu.edu.tw), Yi-Tzu Lee (ytlee@vghtpe.gov.tw).

Table of contents

Clinical adjudication and serum collection

Liquid chromatography conditions setting on LC-HRMS

Parameters for processing the HRMS data using Compound Discoverer

Figure S1. Representative total ion current chromatograms of the 4 acquisition methods.

Figure S2. PCA score plot.

Figure S3-12. MSMS fragmentation and LC chromatography of identified compounds.

Figure S13. Serum levels of 22 metabolites between septic shock and non-septic shock.

Figure S14. Serum levels of 22 metabolites between different disease severity.

Figure S15. Serum levels of 22 metabolites between death and survival groups.

Table S1. MRM transitions of 22 metabolites.

Clinical adjudication and serum collection.

An infectious disease specialist independently determined infection status and final adjudication using standard sepsis and septic shock definitions. Patients enrolled were categorized into four groups according to final adjudication: non-infection, infection without sepsis, sepsis without shock, and septic shock. The description of each group is as follows: (i) non-infectious control, patients without any evidence of infection; (ii) non-septic infection, showing infection evidence (determined using available clinical data 7 days after admission, including culture results, molecular tests, serology tests, relevant imaging, and tissue pathology) but not fulfilling sepsis criteria[1] (acute change in total SOFA score ≥2 points); (iii) non-shock sepsis, presenting infection evidence and meeting sepsis criteria[1] but not fulfilling septic shock criteria[1] (persisting hypotension requiring vasopressors to maintain mean arterial pressure ≥65 mm Hg and having a serum lactate level >2 mmol/L despite adequate volume resuscitation); and (iv) septic shock, patients with sepsis and meeting septic shock criteria[1]. Patients were further divided into a septic shock group (iv) and a non-septic shock group (i, ii, iii). Patients with uncompleted diagnosis were excluded in this study. Physical examination and medical history were recorded at enrollment. Serum sample were obtained during routine clinical testing upon admission before initial treatment. Sera were collected and left to clot at room temperature for 1 h, and then centrifuged at 3000 rpm at 4 °C for 20 min. The supernatant was promptly stored at -80°C before subsequent analysis.

Liquid chromatography conditions setting on LC-HRMS.

Untargeted metabolomics utilized Ultimate 3000 UHPLC coupled with Q Exactive Plus (Thermo Fisher Scientific). Samples were maintained at 4 °C in the autosampler. A cycle run included 1 QC sample per 5 real samples, with a blank run between each sample to prevent carryover.

For separation of polar extracts, an Atlantis HILIC column (3 µm, 2.1 mm x 100 mm, Waters) connected to an Atlantis HILIC VanGuard pre-column (3 µm, 2.1 mm x 5 mm, Waters) was conducted at 30 °C. The mobile solvents included (A) water and (B) acetonitrile/water (90:10 v/v). both containing 0.1% formic acid and 5 mM ammonium formate. Sample injection (2 µL) at a flow rate of 0.5 mL/min followed this gradient: 0-0.5 min, 95%B; 0.5-10 min, 95-50%B; 10-10.1 min, 50-95%B; 10.1-20 min, 95%B.

For separation of nonpolar extracts, a BEH C18 column (1.7 µm, 2.1 mm x 100 mm, Waters) with a BEH C18 VanGuard pre-column (1.7 µm, 2.1 mm x 5 mm, Waters) was conducted at 40 °C. Mobile phase solvents were (A) acetonitrile/water (60:40 v/v) and (B) isopropanol/acetonitrile (90:10 v/v), both containing 0.1% formic acid and 10 mM ammonium formate. Sample injection (10 µL) at a flow rate of 0.25 mL/min followed this gradient: 0-1 min, 40%B; 1-6 min, 40-80%B; 6-11 min, 80-98%B; 11-14 min, 98%B; 14-16 min, 98-40%B; 16-19 min, 40%B.

Parameters for processing the HRMS data using Compound Discoverer.

Metabolites observed with a signal-to-noise ratio (S/N) greater than 10 and peak intensity exceeding 10,000 were detected and grouped based on their mass-to-charge ratio (m/z) with a mass tolerance of less than 5 ppm, and retention time (RT) with a shift of less than 0.5 minutes. Missing values were imputed using the observed minimum in the Fill Gap operation. Peak areas for each metabolite were normalized using quality control (QC) samples to correct for variations resulting from instrument instability. Background signals, defined as showing less than a 5-fold increase compared to blank samples, were removed from the feature list. Metabolites with missing values in more than 10% of the samples were excluded from analysis. A preliminary list of all metabolic features was compiled, including molecular weight, retention time, peak area, and formula prediction.

Figure S1. Method evaluation. Representative total ion current chromatograms of the 4 acquisition methods, including polar ESI+ (A), polar ESI-(B), nonpolar ESI+(C), and nonpolar ESI-(D).

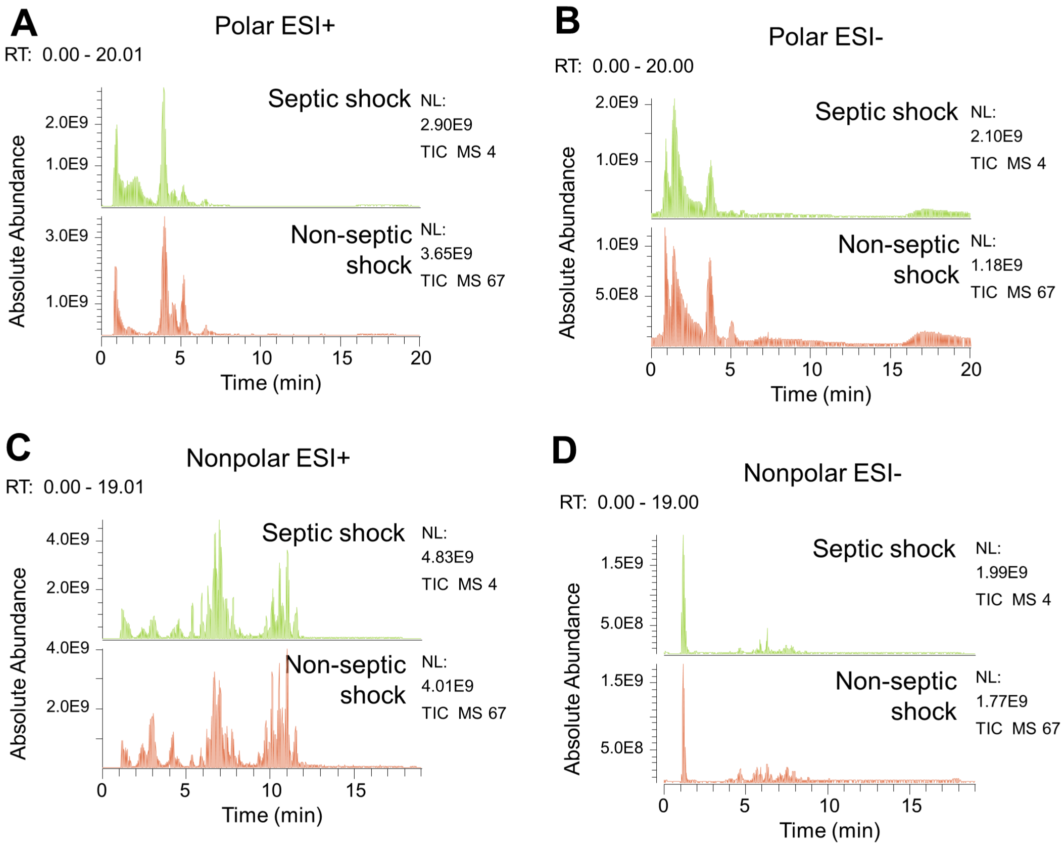

Figure S2. PCA score plot of serum samples from non-septic shock and septic shock patients and QC samples using the all 2892-feature (A) and the 22 discriminative feature (B).

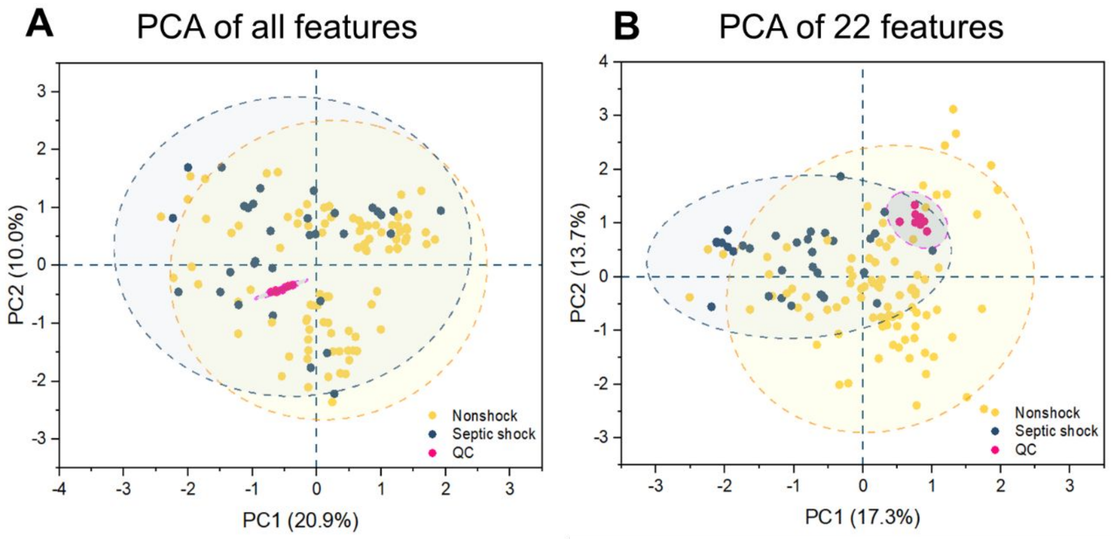

Figure S3. MSMS fragmentation and LC chromatography of ADMA.

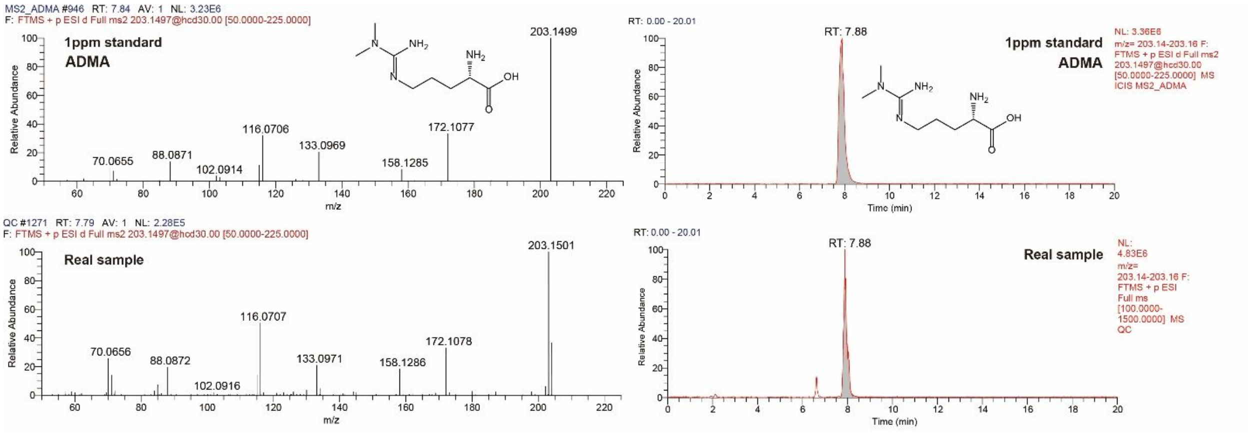

Figure S4. MSMS fragmentation and LC chromatography of N-acetylcadaverine.

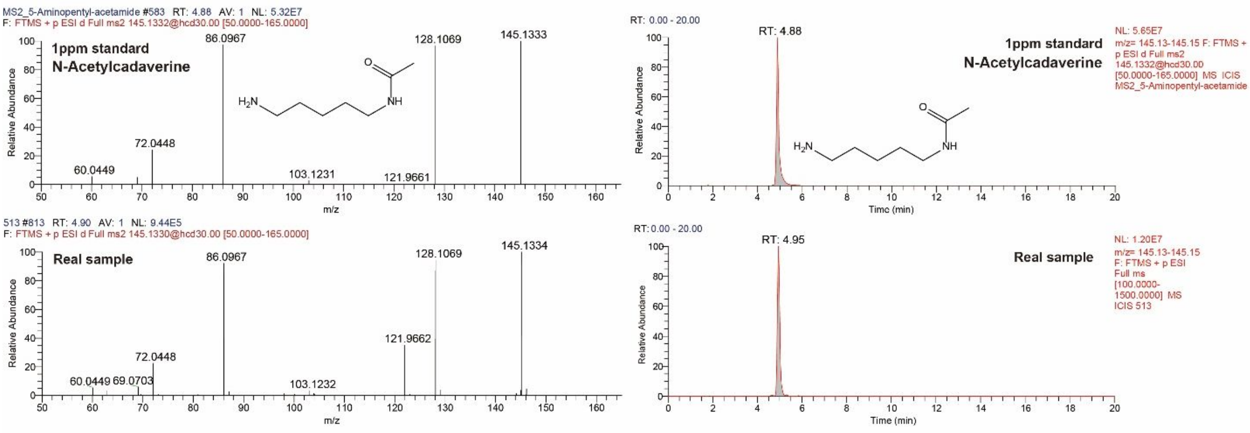

Figure S5. MSMS fragmentation and LC chromatography of hypaphorine.

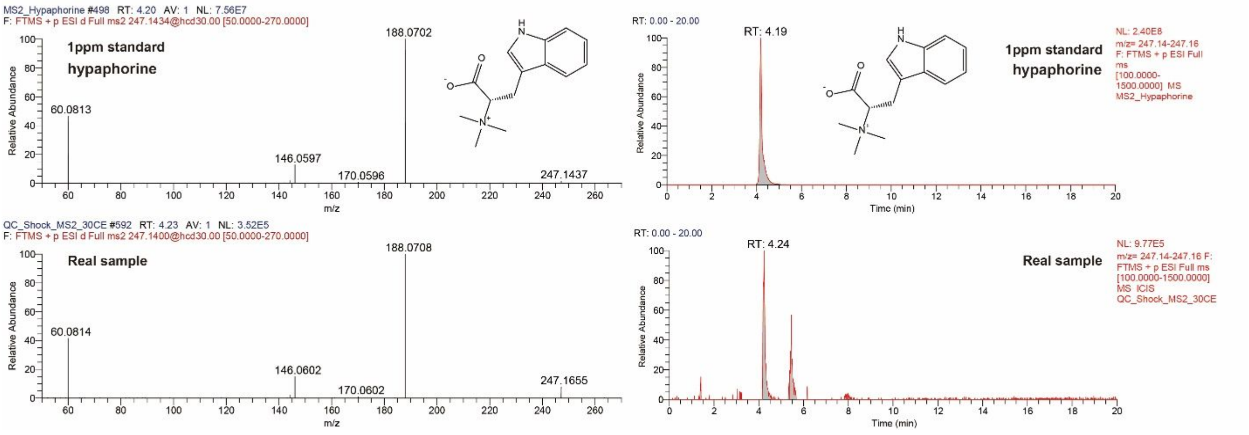

Figure S6. MSMS fragmentation and LC chromatography of linoleyl-carnitine.

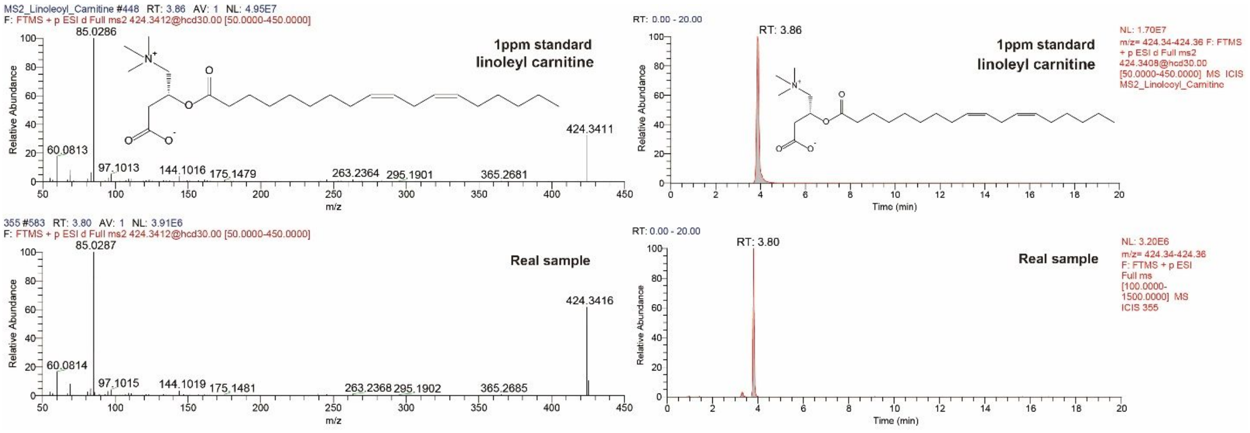

Figure S7. MSMS fragmentation and LC chromatography of 1-methyladenosine.

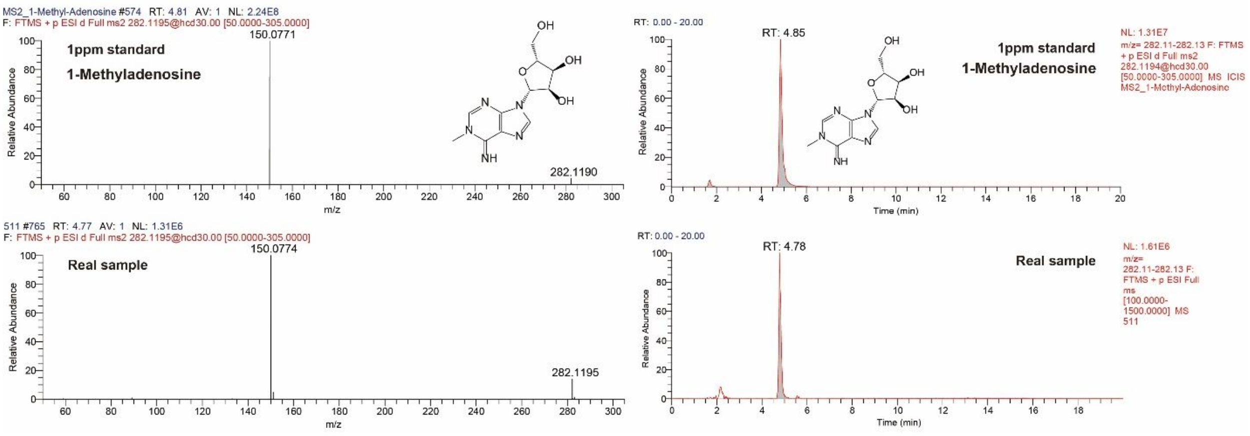

Figure S8. MSMS fragmentation and LC chromatography of DHCer (d18:0/18:0).

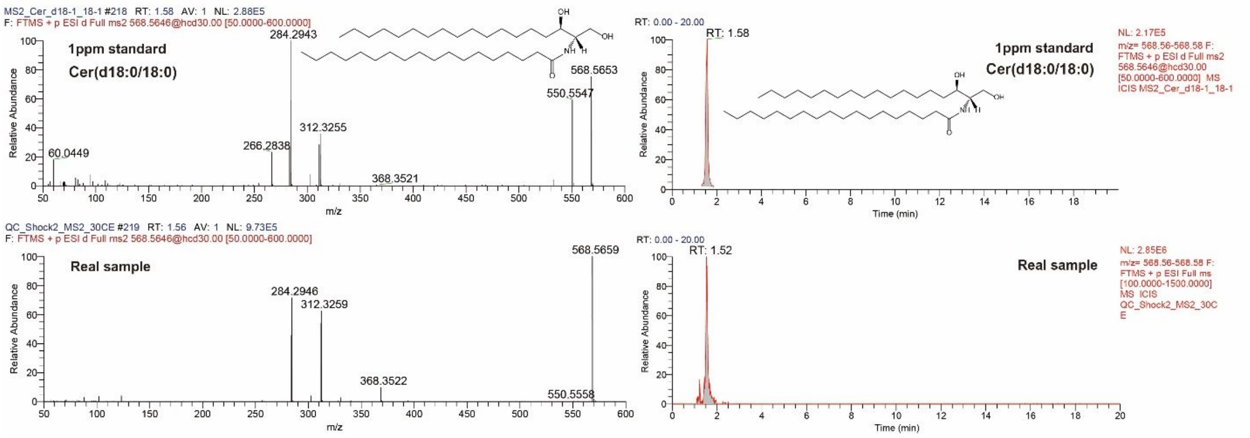

Figure S9. MSMS fragmentation of PC (20:1/0:0).

(online database: https://mona.fiehnlab.ucdavis.edu/spectra/display/LipidBlast057706)

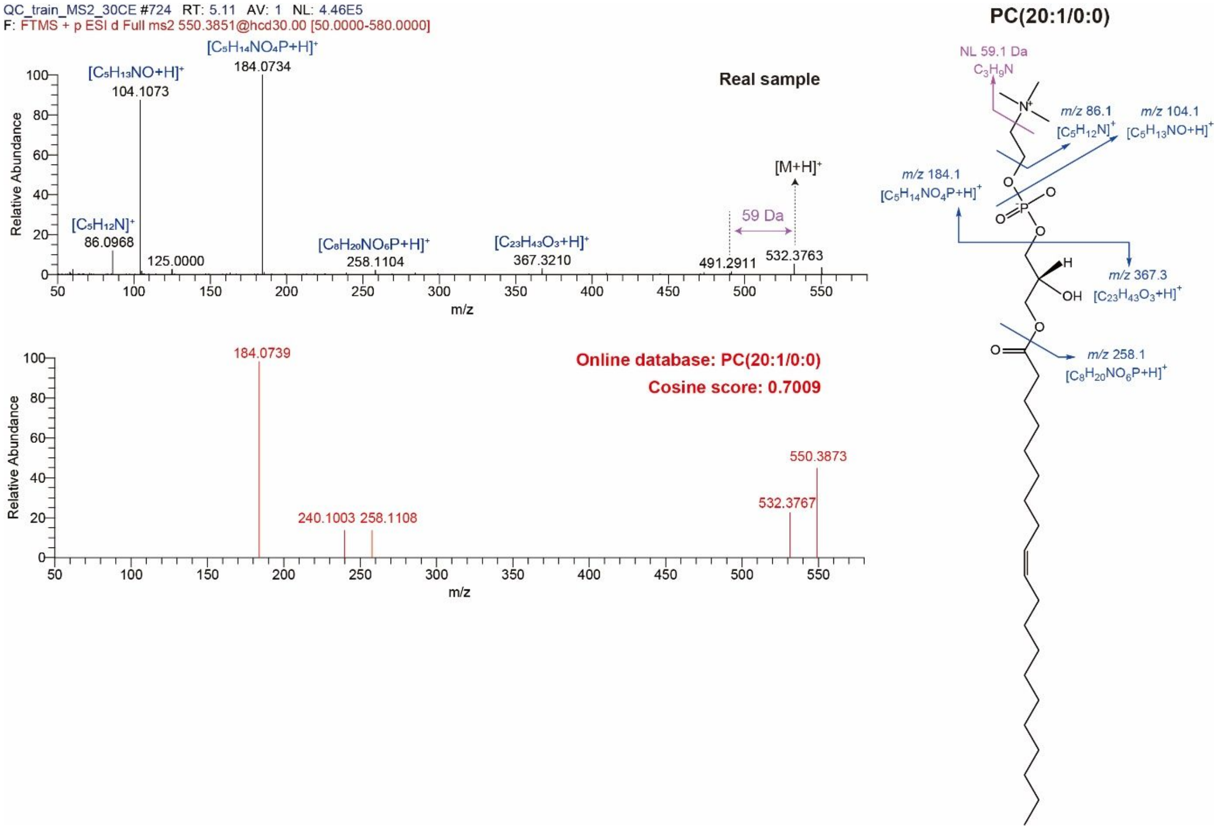

Figure S10. MSMS fragmentation of 5-aminolevulinic acid.

(online database: https://hmdb.ca/spectra/ms_ms/5060)

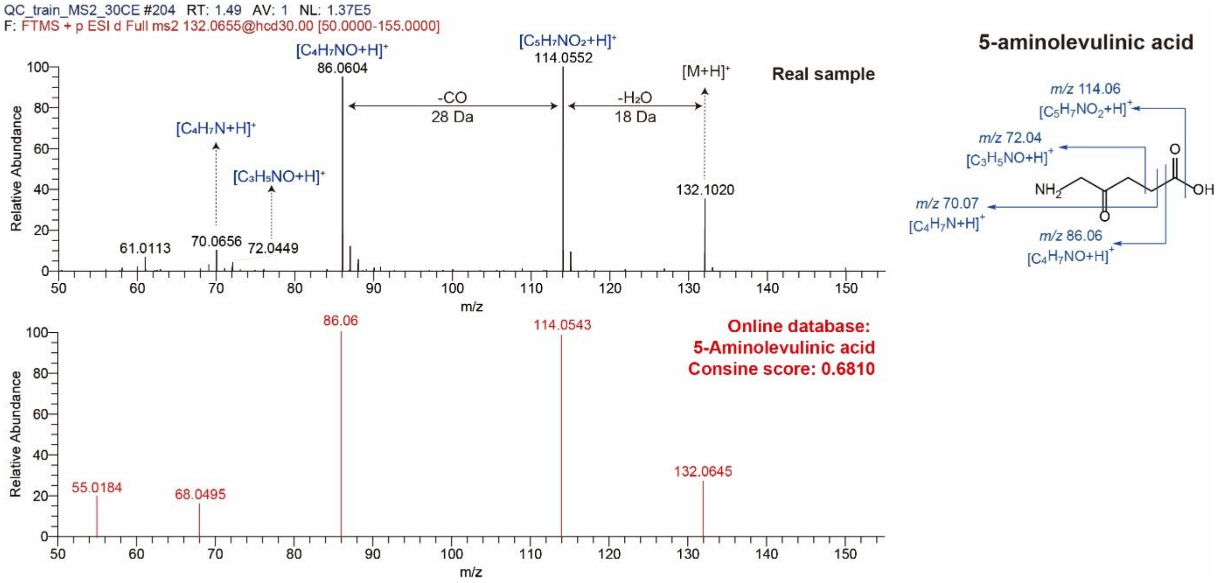

Figure S11. MSMS fragmentation of N-acetyl-D-galactosamine

(online database: https://mona.fiehnlab.ucdavis.edu/spectra/display/EMBL-MCF_spec127081)

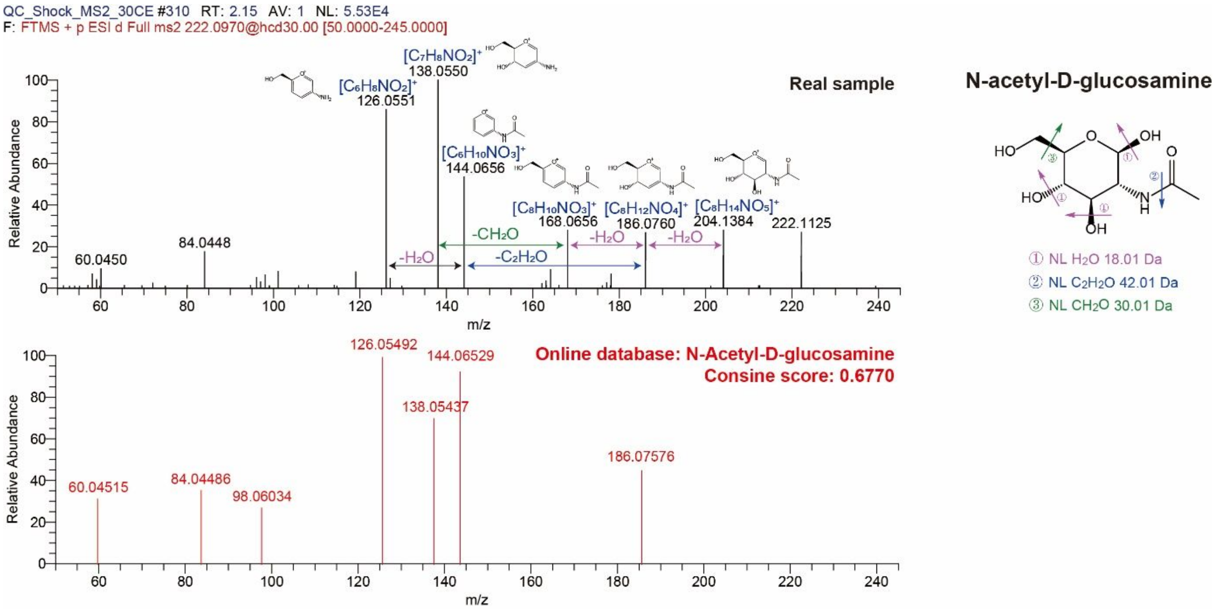

Figure S12. MSMS fragmentation of trigonellinamide.

(online database: https://mona.fiehnlab.ucdavis.edu/spectra/display/PS102702)

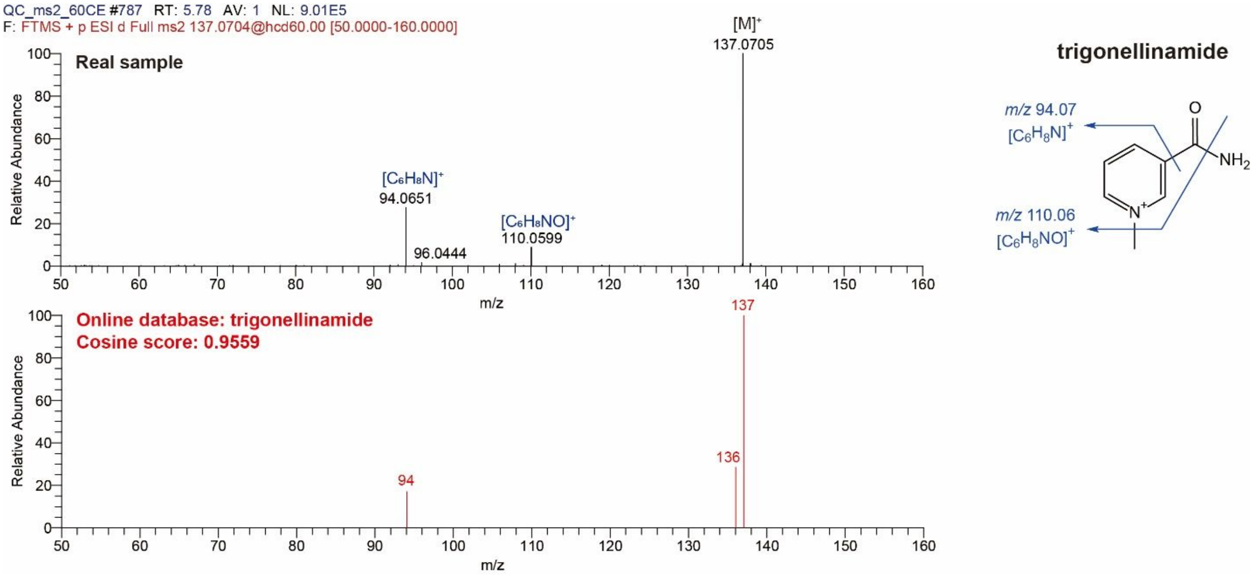

Figure S13. Serum levels of 22 metabolites between septic shock and non-septic shock.

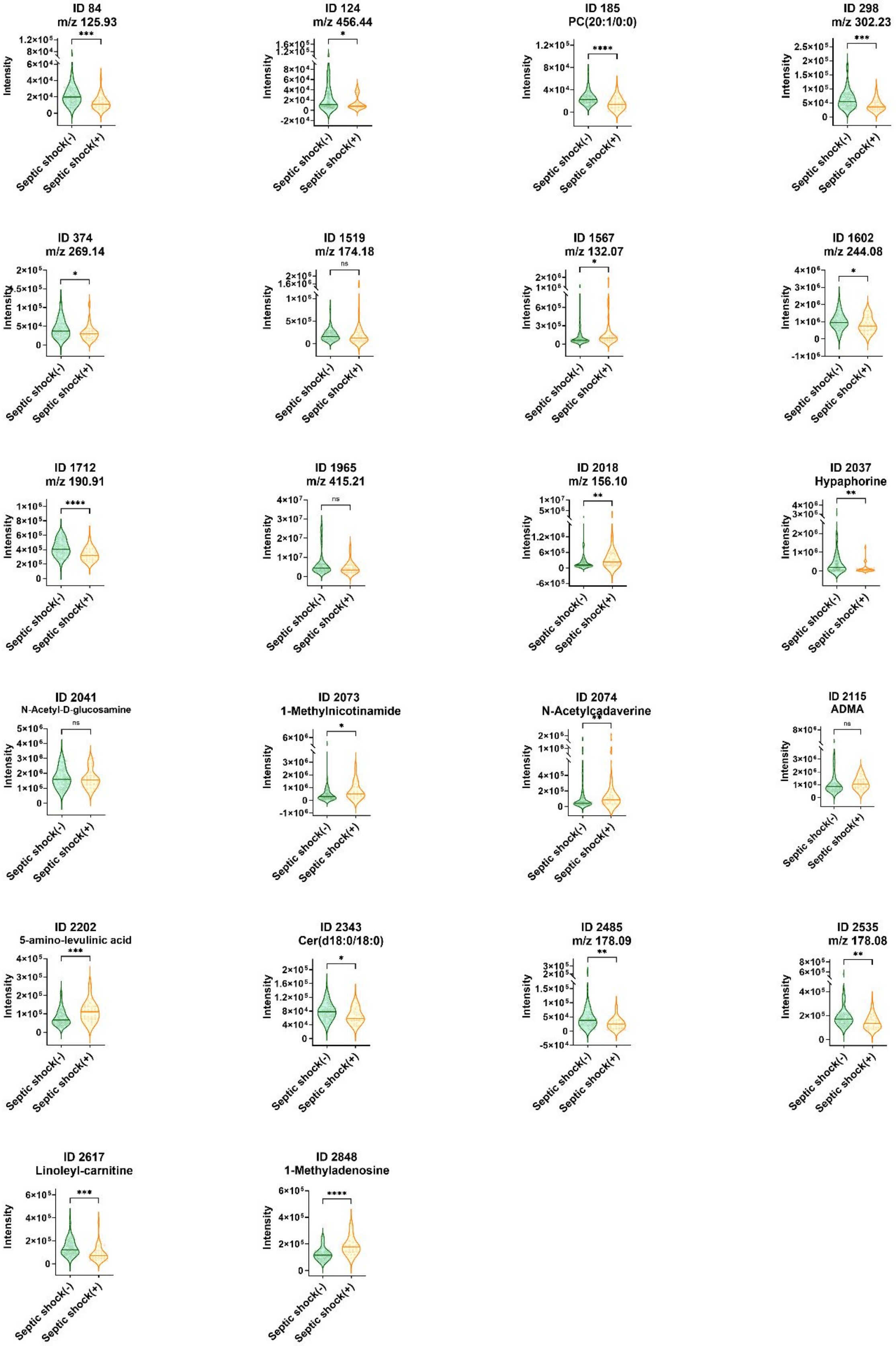

Figure S14. Serum levels of 22 metabolites between different disease severity (control, infection, sepsis and septic shock).

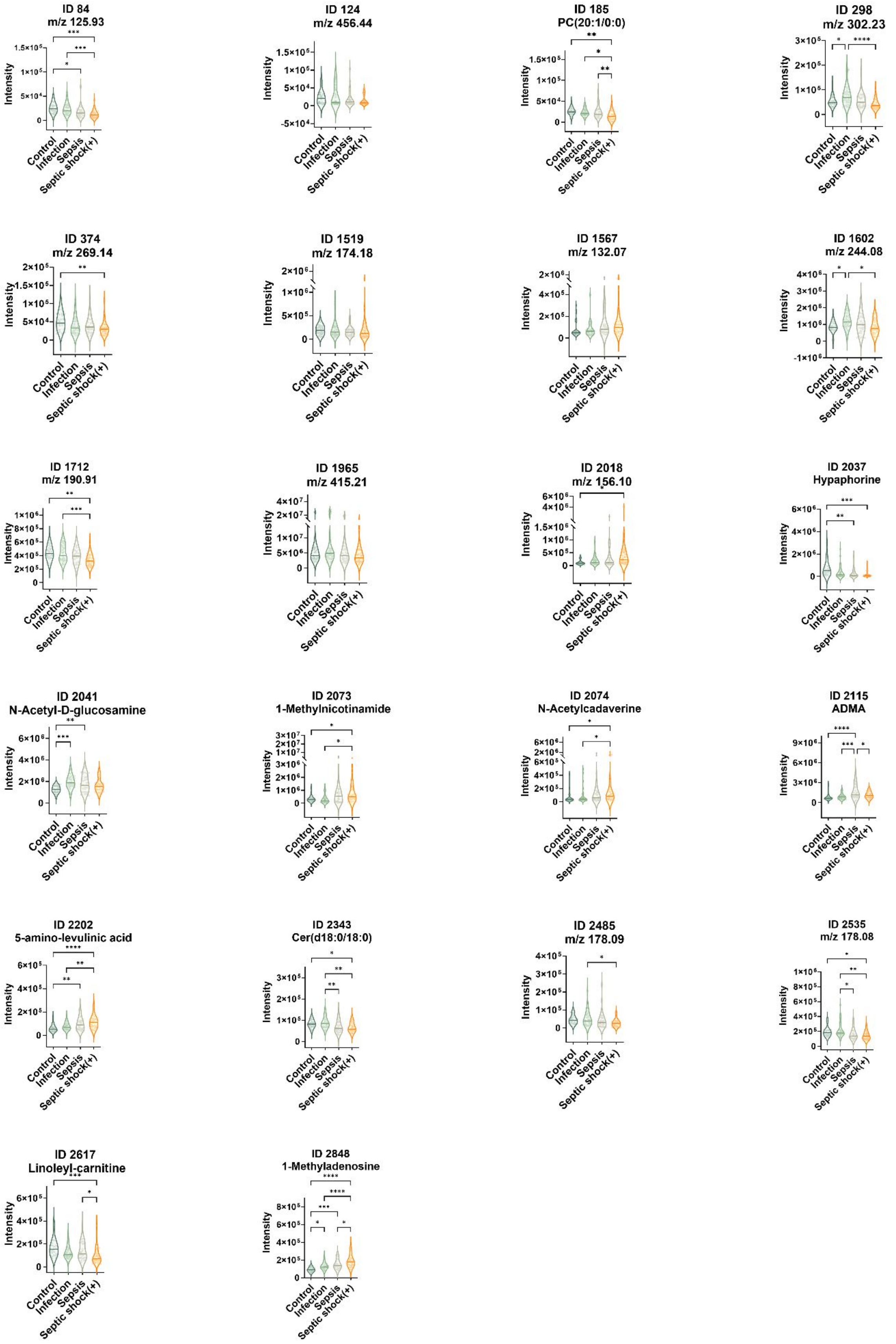

Figure S15. Serum levels of 22 metabolites between death and survival groups.

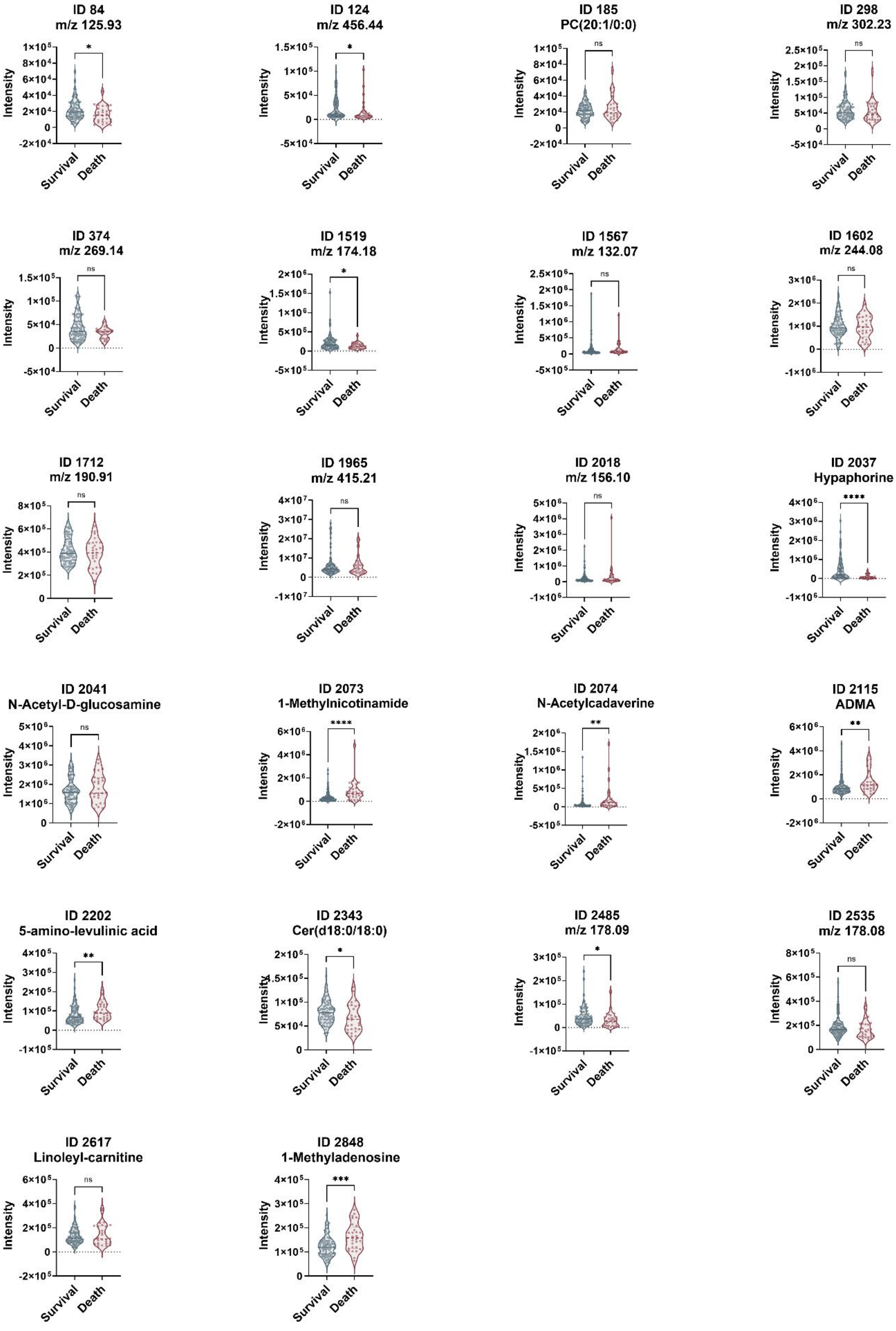

Table S1. MRM transitions of 22 metabolites.

ID	Metabolite	Q1	DP	EP	Q3	CE	CXP
2115	ADMA	203.151	30	12	157	7	15
298	unknown	302.233	30	10	59	15	9
1965	unknown	415.212	30	10	119	15	13
374	unknown	269.139	30	15	137	15	12
1712	unknown	190.912	25	10	123	15	13
2041	N-acetyl-D-galactosamine	222.098	20	10	204	15	10
2617	linoleyl-carnitine	424.342	80	6	85	29	14
2485	unknown	178.087	30	9	132	15	11
185	PC(20:1/0:0)	550.387	30	11	184	35	9
124	unknown	456.441	60	9	200	35	9
2037	hypaphorine	247.144	40	11	188	19	16
2343	Cer(d18:0/18:0)	568.566	20	10	284	40	11
84	unknown	125.938	40	9	125.9	129	129
1519	unknown	174.186	30	13	129	15	10
2202	unknown	132.066	30	9	114	15	9
2848	methyladenosine	282.12	50	7	150	22	18
2535	unknown	105.071	30	9	79	20	9
2018	unknown	174.113	20	9	139	15	9
2073	trigonellinamide	137.071	30	9	94	25	9
1602	unknown	244.08	20	9	203	15	9
2074	N-Acetylcadaverine	145.134	50	7	86	20	10
1567	5-Aminolevulinic acid	132.066	30	10	114	15	9

1. Singer, M.; Deutschman, C. S.; Seymour, C. W.; Shankar-Hari, M.; Annane, D.; Bauer, M.; Bellomo, R.; Bernard, G. R.; Chiche, J.-D.; Coopersmith, C. M.; Hotchkiss, R. S.; Levy, M. M.; Marshall, J. C.; Martin, G. S.; Opal, S. M.; Rubenfeld, G. D.; van der Poll, T.; Vincent, J.-L.; Angus, D. C., The Third International Consensus Definitions for Sepsis and Septic Shock (Sepsis-3). JAMA 2016, 315 (8), 801-810.
